# Synthesis and Characterization of Novel Co(III)/Ru(II) Heterobimetallic Complexes as Hypoxia-Activated Iron-Sequestering Anticancer Prodrugs

**DOI:** 10.3390/molecules29245967

**Published:** 2024-12-18

**Authors:** Tan Ba Tran, Éva Sipos, Attila Csaba Bényei, Sándor Nagy, István Lekli, Péter Buglyó

**Affiliations:** 1 Department of Inorganic & Analytical Chemistry, Faculty of Science & Technology, University of Debrecen, H-4032 Debrecen, Hungary; 2 Department of Pharmacology, Faculty of Pharmacy, University of Debrecen, H-4032 Debrecen, Hungarylekli.istvan@pharm.unideb.hu (I.L.); 3 Department of Physical Chemistry, Faculty of Science & Technology, University of Debrecen, H-4032 Debrecen, Hungary

**Keywords:** organometallic complexes, hypoxia-activated prodrugs, iron chelators, anticancer

## Abstract

Heterobimetallic complexes of an ambidentate deferiprone derivative, 3-hydroxy-2-methyl-1-(3-((pyridin-2-ylmethyl)amino)propyl)pyridin-4(1H)-one (PyPropHpH), incorporating an octahedral [Co(4N)]^3+^ (4N = tris(2-aminoethyl)amine (tren) or tris(2-pyridylmethyl)amine (tpa)) and a half-sandwich type [(η^6^-*p*-cym)Ru]^2+^ (*p*-cym = *p*-cymene) entity have been synthesized and characterized by various analytical techniques. The reaction between PyPropHpH and [Co(4N)Cl]Cl_2_ resulted in the exclusive (O,O) coordination of the ligand to Co(III) yielding [Co(tren)PyPropHp](PF_6_)_2_ (**1**) and [Co(tpa)PyPropHp](PF_6_)_2_ (**2**). This binding mode was further supported by the molecular structure of [Co(tpa)PyPropHp]_2_(ClO_4_)_3_(OH)·6H_2_O (**5**) and [Co(tren)PyPropHpH]Cl(PF_6_)_2_·2H_2_O·C_2_H_5_OH (**6**), respectively, obtained via the slow evaporation of the appropriate reaction mixtures and analyzed using X-ray crystallography. Subsequent treatment of **1** or **2** with [Ru(η^6^-*p*-cym)Cl_2_]_2_ in a one-pot reaction afforded the corresponding heterobimetallic complexes, [Co(tren)PyPropHp(η^6^-*p*-cym)RuCl](PF_6_)_3_ (**3**) and [Co(tpa)PyPropHp(η^6^-*p*-cym)RuCl](PF_6_)_3_ (**4**), in which the piano-stool Ru core is coordinated by the (N,N) chelating set of the ligand. Cyclic voltammetric measurements revealed that the tpa complexes can be reduced at less negative potentials, suggesting their capability to be bioreductively activated under hypoxia (1% O_2_). Hypoxia activation of **2** and **4** was demonstrated by cytotoxicity studies on the MCF-7 human breast cancer cell line. PyPropHpH was shown to be a typical iron-chelating anticancer agent, raising the mRNA levels of TfR1, Ndrg1 and p21. Further qRT-PCR studies provided unambiguous evidence for the bioreduction of **2** after 72 h incubation under hypoxia, in which the characteristic gene induction profile caused by the liberated iron-sequestering PyPropHpH was observed.

## 1. Introduction

A lack of selectivity and the development of resistance are the major drawbacks of Pt(II) complexes currently used in chemotherapy of some cancers [1,2]. To overcome these issues, targeted therapies utilize the differences in the biological properties between cancer and untransformed cells. One such unique property of solid tumors, hypoxia, which develops in poorly vascularized areas, can be exploited by hypoxia-activated anticancer prodrugs [3,4,5]. In this approach, the drug molecules are selectively activated at the site of action, that is, the hypoxic tumor cells.

Co(III) complexes of bioactive ligands with tailored redox potential may serve as promising prodrugs due to the difference in the kinetic behavior between Co(III) and Co(II) forms [6,7,8]. Cancer cells incubated under hypoxia (1% O_2_) have a more negative intracellular redox potential, i.e., a more reductive condition, than those under normoxia (21% O_2_) [9] due to higher expression of reductases and biological reductants [5]. Under low O_2_ concentration, these Co(III) complexes can be reduced to the kinetically labile Co(II) complexes, leading to their dissociation and the liberation of the active ligands from the inactive bound state. In other words, the active drug molecules are selectively released in the hypoxic tumor cells. In addition to Co(III), a second, typically platinum, group metal (PGM) which has proven anticancer potential [2,10,11] can be incorporated to further enhance drug activity.

**Figure 1:**
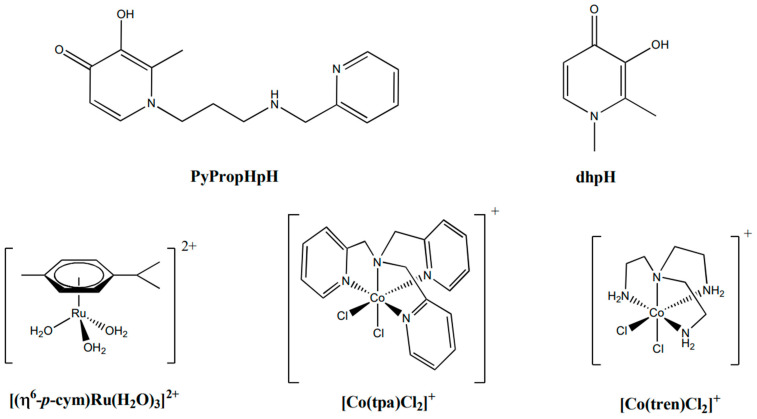
Structural formulas of the studied and model ligands as well as the metal building blocks.

The ligand, PyPropHpH, used in the present work (Figure 1) is a structural modification of dhpH, a well-known, FDA-approved iron chelator used in the treatment of iron overload diseases. Anticancer activity can be expected from strong iron chelators by the depletion of the labile iron pool, inhibition of ribonucleotide reductase or ROS production via redox cycling [12,13]. DhpH has been shown to exhibit moderate in vitro cytotoxicity against various human-derived cancer cell lines [14,15]. Beside the (O,O) donor dhp unit, PyPropHpH also contains an (N,N) chelating set capable of binding soft metal ions such as half-sandwich type organoruthenium or -rhodium ions (Figure 1). Based on detailed solution equilibrium studies, our group has shown that PyPropHpH and structurally related ambidentate ligands can strongly bind Co(III) metal ions via the (O,O) donor set, while the PGM ions exhibit preference toward the (N,N) chelating part of these ligands [16]. The rationale for this design is that the Co(III) core can act as a prodrug moiety of the dhp unit, while the PGM core might enhance the anticancer activity.

The gene induction profile of various iron-chelating agents including dhpH has been extensively characterized by the Richardson group; specifically, TfR1, Ndrg1 and p21^CIP1/WAF1^ (hereafter called p21) mRNA levels increase upon treatment of cancer cells with these agents [17,18,19]. TfR1 plays a crucial role in cellular iron uptake; its mRNA stability increases when cells are deprived of iron [20]. The induction of Ndrg1, which is predominantly reported as a metastatic suppressor protein, is thought to be due to the stabilization of the HIF-1α transcription factor upstream of Ndrg1 during iron depletion [17,21]. The mRNA level of p21, a CDK inhibitor that mediates p53-dependent G1 cell cycle arrest, is elevated by various iron chelators, although the mechanism remains elusive [19]. Based on the well-established qRT-PCR evidence, the aforementioned genes were used as markers of iron depletion in this study.

Herein we report on the synthesis and analytical characterization of novel Co(III) and heterodinuclear Co(III)/Ru(II) complexes of PyPropHpH. The ligand and the complexes were subsequently subjected to cytotoxicity assays on the MCF-7 human breast adenocarcinoma cell line under normoxic (~21% O_2_) and hypoxic (1% O_2_) conditions. The effects of the substances on the expression levels of TfR1, Ndrg1 and p21 genes were also investigated in detail.

## 2. Results and Discussion

### 2.1. Syntheses and Characterization

**Scheme 1:**
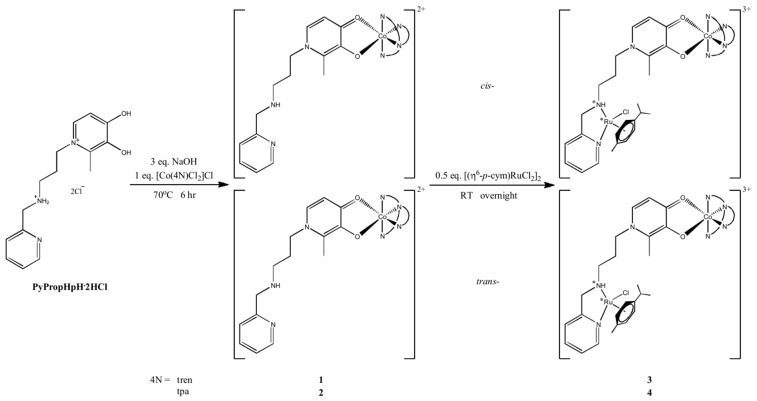
Synthetic procedure of the Co(III) complexes and Co(III)/Ru(II) heterobimetallic complexes. The structures of Co(III)-based geometric isomers are shown. Stereogenic centers are denoted with *.

The general synthetic procedures of the complexes are presented in Scheme 1. Stoichiometric amounts of [Co(4N)Cl_2_]Cl (4N = tren or tpa) precursors and PyPropHpH·2HCl were reacted in water in the presence of three equivalent bases at 70 °C for 6 h to obtain the two Co(III) complexes. Without isolation of the Co(III) complexes, one-pot reactions with [(η^6^-*p*-cym)RuCl_2_]_2_ at RT overnight were carried out to form the respective heterobimetallic complexes. For all cases, PF_6_^–^ as the bulky anion was used to isolate the colored products.

The ^1^H-NMR spectra of the four complexes showed resonance signals with the expected integrals and number of isomers. The spectra of **1** and **2** indicated the existence of two geometric isomers, as depicted in Scheme 1, at a roughly 1:1 ratio (Figures S1–S3). For **1**, the doubling due to isomerization at the octahedral Co center can only be observed for signals belonging to the pyridinone ring (Figures S1 and S2), which includes two ring protons in the aromatic region and a methyl proton at ~2.5 ppm (for the ^1^H-NMR measurement in D_2_O). In the case of **2**, the doubling due to isomerization could be detected for all protons due to the bulkiness of tpa (Figure S3). The pyridinone ring protons and the methyl proton, which are closest to the bulky tpa ligand, each showed two well-separated signals of the *cis*- and *trans*-isomers.

To rule out any solvolysis and/or dissociation of the heterobimetallic complexes, **3** and **4**, (Figures S4–S6) the ^1^H-NMR spectra were registered both in DMSO-d^6^ and acetone-d^6^. For **3**, the almost identical spectra shown in Figures S5 and S6 indicated no change in the presence of DMSO. The results also demonstrated the presence of four distinct isomers in a ratio of approximately 3:3:1:1 both in acetone and in DMSO. Upon chelation with Ru(II), the pseudo-octahedral Ru core and the secondary amine nitrogen of PyPropHpH become stereogenic centers (Scheme 1) and hence enantiomers and diastereomers can be formed in addition to geometric isomerization at the Co center. Considering the two geometric isomers at the Co unit, altogether eight isomers can be formed; however, since they form enantiomeric pairs (see Figure S7) that are non-distinguishable in the NMR, four signals for each hydrogen can be assumed in the spectra. Although the assignment was complicated by the doubling due to isomerization and overlapping with the solvent peaks, for **3** most protons were identified with the expected integrals. In the case of **4**, the ^1^H-NMR spectra demonstrated the assumed 1:4:6 ratio of one of the pyridinone ring proton of PyPropHpH, the ring protons of *p*-cym and the -CH_2_ protons of the 4N tripodal ligand, proving that the one-pot reaction afforded adequately pure heterobimetallic complexes. As a further piece of evidence, the COSY spectrum of **3** is shown in Figure S8 while the four spin systems belonging to the above four isomers are demonstrated in Figure S9 for the various hydrogens. Similar isomer formation was found in the [(η^5^-Cp*)Rh]^2+^–PyPropHp system at a 2:1 metal ion to ligand molar ratio for the major dinuclear complex featuring both (O,O) and (N,N) coordination of the metal ion [16].

ESI-TOF-MS analysis in positive mode provided further proof for the identity of the complexes. As an example, the spectra of **3** and **4** (Figure S10) displayed a characteristic pattern due to the abundance of multiple isotopes of Ru. All the identified cations are summarized in Table S1. Of note, Co(II) species of **2** and **4** were detected, indicating Co reduction of the tpa complexes under MS conditions.

**Figure 2:**
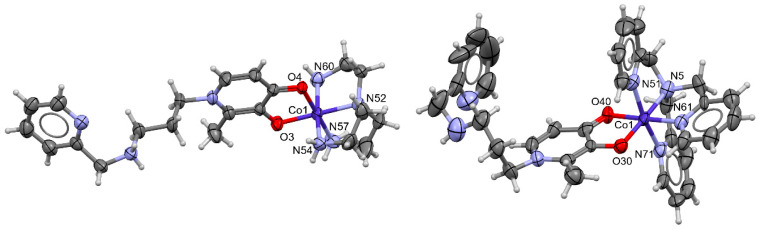
Ortep view of the molecular structure of [Co(tren)(PyPropHpH)]^3+^ (**top**) and [Co(tpa)(PyPropHp)]^2+^ (**bottom**) cations. The anions and solvent molecules are omitted for clarity.

Single crystals incorporating the [Co(tren)(PyPropHpH)]^3+^ or [Co(tpa)(PyPropHp)]^2+^ cations could be obtained via the slow evaporation of aqueous or ethanolic solutions at 4 °C. The X-ray studies unambiguously confirmed the coordination of Co(III) entities to the (O,O) chelating set of PyPropHpH (Figure 2). Both the Co(III) complexes were crystallized as the *trans*-isomer, in which the tertiary amine of the 4N ligand is in the *trans*-position to the deprotonated hydroxyl group of PyPropHpH.

### 2.2. Electrochemical Studies

CV studies provided useful information about the redox properties of the complexes. The measurements were carried out by reducing the potential from 100 to −1200 mV and then increasing it back to 100 mV. PyPropHpH and Ru(II) were found to be redox-inactive within this range; therefore, the resulting voltammetric peaks of the complexes only correspond to the redox process of Co. All four complexes displayed irreversible reductions of Co(III). This suggests that the formed Co(II) complexes are kinetically labile and would dissociate to release PyPropHpH. The reduction potentials of the tpa complexes are less negative than those of the tren complexes (Table 1), meaning the tpa complexes are more reducible. This is because of the *π*-back-bonding capability of tpa that stabilizes the generated Co(II) complexes, driving the equilibrium toward the reduced form. This property is in accordance with other previously studied Co(III) complexes [22,23]. Since the reduction potential range of cellular reductases is estimated to be within −200 to −400 mV vs. NHE [9,24,25], the tpa complexes might be selectively reduced in tumor cells. On the contrary, the tren complexes would probably remain inert in biological systems.

### 2.3. Stability Testing

The UV–Vis spectra of the complexes in PBS at RT did not change significantly over the studied duration (Figure S11). No precipitation was detected. It was concluded that the complexes are likely stable in biological systems.

### 2.4. Lipophilicity Measurements

All complexes displayed relatively high hydrophilicity, with log D < −1.8 (Table 2), hinting toward low cellular uptake by passive diffusion of these compounds [26].

### 2.5. Cytotoxicity Studies

**Figure 3:**
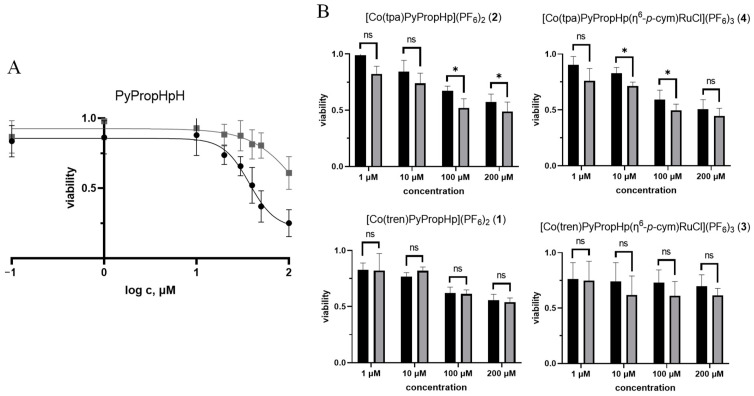
(**A**) Cell viability after 72 h treatment of PyPropHpH under normoxia (black) and hypoxia (gray). (**B**) Cell viability after 72 h treatment with the complexes under normoxia (black) and hypoxia (gray). Data points are presented as mean (SD). Multiple paired *t* test and Holm–Šidák post hoc test were used to analyze the data. Significance level: ns: *p* > 0.05, *: *p* ≤ 0.05, **: *p* ≤ 0.01, ***: *p* ≤ 0.001, ****: *p* ≤ 0.0001.

The antiproliferative effects of the four complexes and PyPropHpH on the breast cancer MCF-7 cell line under both normoxia (~21% O_2_) and hypoxia (1% O_2_) were measured by MTT assays. PyPropHpH was found to be moderately cytotoxic under normoxia with an IC_50_ of 40 μM (95% CI: 35 to 46). However, PyPropHpH was less active under hypoxia (Figure 3A), similar to several structurally related flavonoid ligands in a previous study [23]. Many compounds lose their cytotoxicity under hypoxia due to the emergence of various tumor drug resistance mechanisms that are induced at low oxygen levels [27]; however, there are currently no data on the cytotoxicity of other iron-chelating agents at 1% O_2_.

All four complexes displayed low potency and hence it was not worth determining the accurate IC_50_ values; only four concentrations (1, 10, 100 and 200 μM) were tested. As predicted by the CV studies, the tpa complexes demonstrated statistically significant activation under hypoxia while the tren complexes did not (Figure 3B). The IC_50_ values of both **2** and **4** were estimated to be ~100 μM under hypoxia. We hypothesize that although the Co reduction leads to the release of the iron-chelating dhp unit of PyPropHpH, the cancer cells were not sensitive to PyPropHpH under hypoxia. The incorporation of Ru(II) neither enhanced nor disturbed the cytotoxic activity and hypoxia activation of the Co core. The lack of enhanced cytotoxicity by the Ru moiety is probably due to the low lipophilicity of the complexes, which correlates with low cell membrane penetration [26].

Further dynamic studies of **2** and **4** revealed that hypoxia activation was not achieved after 24 or 48 h of treatment (Figures S12 and S13). It seems that one-electron reduction of Co(III) at 1% O_2_ is a slow process.

### 2.6. Gene Expression Analysis

**Figure 4:**
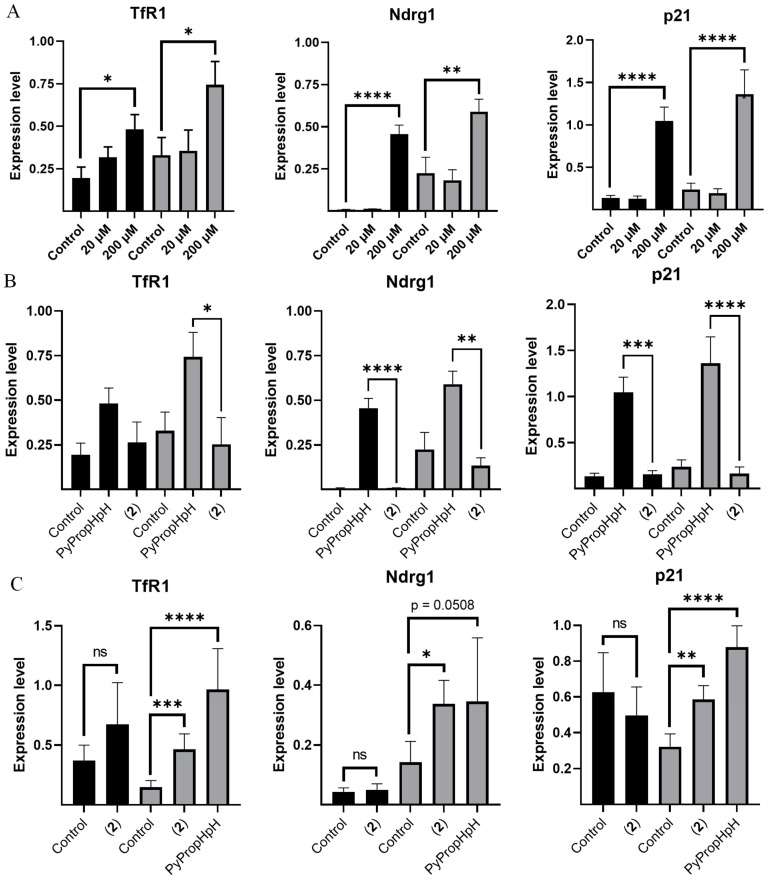
Gene expression level changes calculated by 2^40-ct^ method after (**A**) 24 h treatment with PyPropHpH, (**B**) 24 h treatment with PyPropHpH and **2** at 200 μM and (**C**) 72 h treatment with PyPropHpH and **2** at 100 μM under normoxia (black) and hypoxia (gray). Data points are presented as mean (SD). Two-way ANOVA for (**A**,**B**) and one-way ANOVA for (**C**) followed by Dunett’s post hoc test for (**A**) and Tukey’s post hoc test for (**B**,**C**) were used to analyze the data. Significance level: ns: *p* > 0.05, *: *p* ≤ 0.05, **: *p* ≤ 0.01, ***: *p* ≤ 0.001, ****: *p* ≤ 0.0001.

The qRT-PCR studies revealed that PyPropHpH produced the gene induction profile characteristic of cells treated by iron chelators (Figure 4A). The induction of TfR1, Ndrg1 and p21 was still observed under hypoxia, meaning the iron-sequestering effect of PyPropHpH persisted. This might exclude metabolic inactivation and efflux pumps as the mechanisms of tumor resistance against PyPropHpH under hypoxia.

Complex **2** was selected to investigate its hypoxia activation using qRT-PCR. It was found that the incorporation of Co(III) to the iron-binding site of PyPropHpH effectively prevented the induction of TfR1, Ndrg1 and p21 genes (Figure 4B). This proved that the Co(III) complex principally works as an inactive prodrug under normoxia. Hypoxia activation and liberation of PyPropHpH were not observed after 24 h treatment, as indicated by the absence of the induction of TfR1, Ndrg1 and p21 genes (Figure 4B). This is consistent with the viability studies with a 24 h treatment duration (Figure S12).

As PyPropHpH is not cytotoxic under hypoxia, it was possible to perform gene expression analysis of MCF-7 cells treated with 100 μM of PyPropHpH for 72 h under hypoxic conditions. Similarly to the 24 h incubation study, the iron-chelating effect of PyPropHpH could be observed (Figure 4C). It was shown that 100 μM of **2** increased the mRNA levels of the three genes under hypoxia but not under normoxia (Figure 4C), which confirmed that **2** acts as a prodrug under normoxia and releases PyPropHpH under hypoxia.

## 3. Materials and Methods

### 3.1. Chemicals

The CoCl_2_∙6H_2_O, NaNO_2_, tris(2-aminoethyl)amine (tren), tris(2-pyridylmethyl)-amine (tpa), NH_4_PF_6_, NaClO_4_∙H_2_O, tetra-n-butyl-ammonium fluoride (TBAF), [(η^6^-*p*-cym)RuCl_2_]_2_, NaOH, D_2_O, DMSO-*d*^6^, acetonitrile, ethanol, n-octanol and diethyl ether were commercial products from Merck (Darmstadt, Germany), Sigma-Aldrich (St. Louis, MO, USA), TCI Chemicals (Tokyo, Japan), VWR (Radnor, PA, USA), Acros Organics (Geel, Belgium), Scharlau (Barcelona, Spain) or Reanal (Budapest, Hungary) and used as received. The [Co(tren)(NO_2_)_2_]Cl, [Co(tren)Cl_2_]Cl·H_2_O, [Co(tpa)(NO_2_)_2_]Cl, [Co(tpa)Cl_2_]Cl·2H_2_O [28] and PyPropHpH∙2HCl [16] were synthesized and purified according to literature procedures. All complexes in the present study were >95% pure by elemental analysis. Elemental analysis (C, H, N) was conducted on an Elementar Variomicro Cube instrument at the Department of Organic Chemistry, University of Debrecen.

### 3.2. Syntheses

#### 3.2.1. [Co(tren)PyPropHp](PF_6_)_2_ (**1**)

PyPropHpH∙2HCl (315.2 mg, 0.91 mmol) was dissolved in 5 mL water and NaOH (109.2 mg, 2.73 mmol) was added. [Co(tren)Cl_2_]Cl·H_2_O (300.0 mg, 0.91 mmol) was added and the purple reaction mixture was stirred at 70 °C for 6 h. After cooling, NH_4_PF_6_ (296.8 mg, 1.82 mmol) was added. The solution was filtered and brought to 1 mL under diminished pressure. The solution was stood at 4 °C overnight, resulting in a purple oil. The oil was scratched with the repeated addition of cold water until a pinkish powder formed. The solid was filtered off, washed with 96% ethanol and ether and dried in vacuo. Yield: 357.8 mg (51%). Ratio of the isomers A:B = 1:1. ^1^H NMR (500 MHz, D_2_O): *δ*/ppm = 8.60 (d, 1H, pyridine, isomer A+B), 7.94 (t, 1H, pyridine, isomer A+B), 7.61–7.48 (m, 3H, Ar-H, isomer A+B), 6.84, 6.74 (2 d, 1H, pyridinone, isomer A+B), 4.37 (s, 2H, -CH_2_, isomer A+B), 4.29 (m, 2H, -CH_2_, isomer A+B), 3.77–3.61 (m, 2H, -CH_2_ tren, isomer A+B), 3.43–3.33 (m, 2H, -CH_2_ tren, isomer A+B), 3.24–3.13 (m, 6H, -CH_2_ tren and -CH_2_, isomer A+B), 3.00–2.89 (m, 4H, -CH_2_ tren, isomer A+B), 2.60, 2.51 (2 s, 3H, -CH_3_, isomer A+B), 2.26 (m, 2H, -CH_2_, isomer A+B). ^1^H NMR (500 MHz, DMSO-*d*^6^): *δ*/ppm = 8.65 (d, 1H, Ar-H pyridine, isomer A+B), 7.91 (t, 1H, Ar-H pyridine, isomer A+B), 7.67 (m, 1H, Ar-H pyridinone, isomer A+B), 7.54 (d, 1H, Ar-H pyridine, isomer A+B), 7.45 (t, 1H, Ar-H pyridine, isomer A+B), 6.74, 6.64 (2 d, 1H, Ar-H pyridinone, isomer A+B), 5.37 (m, 2H, -NH_2_ tren, isomer A+B), 4.98 (m, 2H, -NH_2_ tren, isomer A+B), 4.70 (m, 2H, -NH_2_ tren, isomer A+B), 4.32 (s, 2H, -CH_2_,isomer A+B), 4.22 (t, 2H, -CH_2_, isomer A+B), 3.21–2.72 (m, 14H, -CH_2_ tren and -CH_2_, isomer A+B), 2.50, 2.46 (2 s, 3H, -CH_3_, isomer A+B), 2.09 (m, 2H, -CH_2_, isomer A+B). Elem. anal. calcd for C_21_H_36_N_7_O_2_CoP_2_F_12_·0.4NaPF_6_·1.2H_2_O, %: C, 29.46; H, 4.52; N, 11.45. Found, %: C, 29.07; H, 4.89; N, 11.35. HR-MS (ESI, positive ion): *m*/*z* = 238.6123 (C_21_H_36_N_7_O_2_Co: 238.6126; [Co(tren)(PyPropHp)]^2+^).

#### 3.2.2. [Co(tpa)PyPropHp](PF_6_)_2_ (**2**)

PyPropHpH∙2HCl (70.7 mg, 0.203 mmol) was dissolved in 5 mL water and NaOH (24.6 mg, 0.615 mmol) was added. [Co(tpa)Cl_2_]Cl·2H_2_O (100.0 mg, 0.203 mmol) was added and the purple reaction mixture was stirred at 70 °C for 6 h. The solution was filtered. After cooling, NH_4_PF_6_ (66.3 mg, 0.406 mmol) was added, resulting in the formation of a purple microcrystalline precipitate. The solution was stood at 4 °C overnight for further precipitation. The solid was filtered off, washed with cold water, cold abs. ethanol and ether and dried in vacuo. Yield: 69.5 mg (37%). Ratio of the isomers A:B = 1:1. ^1^H NMR (500 MHz, DMSO-*d*^6^): *δ*/ppm = 9.21, 9.13 (2 d, 1H, Ar-H, isomer A+B), 8.63, 8.59 (2 d, 1H, Ar-H, isomer A+B), 8.38, 8.33 (2 d, 2H, Ar-H, isomer A+B), 8.10–7.39 (m, 13H, Ar-H), 7.17, 6.52 (2 d, 1H, Ar-H, isomer A+B), 5.51–5.07 (m, 6H, -CH_2_ tpa, isomer A+B), 4.31–4.13 (m, 4H, -CH_2_, isomer A+B), 3.01–2.92 (m, 2H, -CH_2_, isomer A+B), 2.96, 2.33 (2 s, 3H, -CH_3_, isomer A+B), 2.10, 1.98 (2 p, 2H, -CH_2_, isomer A+B). Elem. anal. calcd for C_33_H_36_N_7_O_2_CoP_2_F_12_·0.7NaPF_6_, %: C, 38.51; H, 3.53; N, 9.53. Found, %: C, 38.68; H, 3.54; N, 9.41. HR-MS (ESI, positive ion): *m*/*z* = 310.6128 (C_33_H_36_N_7_O_2_Co: 310.6126; [Co(tpa)(PyPropHp)]^2+^).

#### 3.2.3. [Co(tren)PyPropHp(η^6^-p-cym)RuCl](PF_6_)_3_ (**3**)

PyPropHpH∙2HCl (105.1 mg, 0.303 mmol) was dissolved in 3 mL water and NaOH (36.4 mg, 0.909 mmol) was added. [Co(tren)Cl_2_]Cl·H_2_O (100.0 mg, 0.303 mmol) was added and the purple reaction mixture was stirred at 70 °C for 6 h. After cooling, the solution was added to previously suspended [(η^6^-*p*-cym)RuCl_2_]_2_ (92.9 mg, 0.152 mmol) in 3 mL water and the brown mixture was stirred overnight at RT. The solution was filtered. NH_4_PF_6_ (148.4 mg, 0.910 mmol) was added, resulting in a light brown microcrystalline precipitate. The solution was stood at 4 °C overnight for further precipitation. The solid was filtered off, washed with cold water and air-dried by grinding and letting it stand on the vacuum filter for at least 5 min. It was then washed with cold abs. ethanol and ether and dried in vacuo. Yield: 97.1 mg (27%). Ratio of the isomers A:B:C:D = 3:3:1:1. ^1^H NMR (500 MHz, DMSO-*d*^6^): *δ*/ppm = 9.22, 9.20 (2 d, Ar-H, isomer C+D), 9.04 (d, Ar-H pyridine, isomer A+B), 8.05 (t, Ar-H pyridine, isomer A+B), 8.00 (t, Ar-H pyridine, isomer C+D), 7.76, 7.74 (2 d, Ar-H pyridinone, isomer A+B), 7.69–7.48 (m, Ar-H pyridine, isomer A+B+C+D, Ar-H pyridinone, isomer C+D), 6.78, 6.68, (2 d, Ar-H pyridinone, isomer A+B), 6.75, 6.65 (2 d, Ar-H pyridinone, isomer C+D), 5.96–5.77 (m, 4H, Ar-H *p*-cym, isomer A+B+C+D), 5.24–2.06 (m, -NH_2_ and -CH_2_ tren, -CH_2_, -CH *p*-cym, isomer A+B+C+D) 2.06, 2.04 (2 s, -CH_3_ *p*-cym, isomer C+D), 1.94, 1.93 (2 s, -CH_3_ *p*-cym, isomer A+B), 1.23–1.03 (m, -CH_3_ *i*-Pr, isomer A+B+C+D). Elem. anal. calcd for C_31_H_50_N_7_O_2_ClCoRuP_3_F_18_·0.8NaPF_6_, %: C, 28.26; H, 3.83; N, 7.44. Found, %: C, 27.91; H, 3.85; N, 7.84. HR-MS (ESI, positive ion): *m*/*z* = 249.4019 (C_31_H_50_N_7_O_2_ClCoRu: 249.4025; [Co(tren)(PyPropHp)(η^6^-*p*-cym)RuCl]^3+^).

#### 3.2.4. [Co(tpa)PyPropHp(η^6^-p-cym)RuCl](PF_6_)_3_ (**4**)

PyPropHpH∙2HCl (70.4 mg, 0.203 mmol) was dissolved in 5 mL water and NaOH (24.4 mg, 0.609 mmol) was added. [Co(tpa)Cl_2_]Cl·2H_2_O (100.0 mg, 0.203 mmol) was added and the purple reaction mixture was stirred at 70 °C for 6 h. After cooling, the solution was added to previously suspended [(η^6^-*p*-cym)RuCl_2_]_2_ (62.3 mg, 0.102 mmol) in 5 mL water and the brown mixture was stirred overnight at RT. The solution was filtered. NH_4_PF_6_ (99.4 mg, 0.610 mmol) was added, resulting in a brown precipitate. The solution was stood at 4 °C overnight for further precipitation. The solid was filtered off, washed with cold water, 96% ethanol and ether and dried in vacuo. Yield: 188.1 mg (70%). Ratio of the isomers A:B:C:D = 2:2:1:1. ^1^H NMR (500 MHz, DMSO-*d*^6^): *δ*/ppm = 9.22, 9.14 (2 d, Ar-H, isomer A+B), 9.22–9.13 (m, Ar-H, isomer C+D), 9.04, 9.01 (2 d, Ar-H, isomer A+B), 8.39–7.38 (m, Ar-H pyridine, isomer A+B+C+D), 7.19, 6.54 (2 d, Ar-H pyridinone, isomer A+B), 7.18, 6.49 (2 d, Ar-H pyridinone, isomer C+D), 5.93–5.66 (m, Ar-H *p*-cym, isomer A+B+C+D), 5.52–5.08 (m, -CH_2_ tpa, isomer A+B+C+D), 4.57–4.03 (m, -CH_2_, isomer A+B+C+D), 3.55–2.03 (m, -CH_2_, -CH *p*-cym, isomer A+B+C+D), 2.99, 2.37 (2 s, -CH_3_, isomer A+B), 2.98, 2.36 (2 s, -CH_3_, isomer C+D), 2.01, 1.94 (2 s, -CH_3_, isomer C+D), 1.89, 1.82 (2 s, -CH_3_, isomer A+B), 1.10–0.91 (8 d, -CH_3_ *i*-Pr, isomer A+B+C+D). Elem. anal. calcd for C_43_H_50_N_7_O_2_ClCoRuP_3_F_18_, %: C, 38.91; H, 3.80; N, 7.39. Found, %: C, 38.62; H, 4.02; N, 7.31. HR-MS (ESI, positive ion): *m*/*z* = 297.4024 (C_43_H_50_N_7_O_2_ClCoRu: 297.4025; [Co(tpa)(PyPropHp)(η^6^-*p*-cym)RuCl]^3+^).

### 3.3. NMR

NMR measurements were carried out using a Bruker Avance I 500 MHz NMR spectrometer (Bruker, Billerica, MA, USA) at RT on samples prepared in D_2_O, DMSO-*d*^6^ or acetone-*d^6^*. Calibration was performed using the residual solvent signals (D_2_O: 4.79 ppm; DMSO-*d*^6^: 2.50 ppm, acetone-*d*^6^: 2.05 ppm). The spectra were evaluated with MestReNova v9.0.1 software.

### 3.4. ESI-MS

High-resolution ESI-TOF MS measurements in positive mode were carried out on a Bruker maXis II UHR ESI-TOF MS instrument (Bruker, Billerica, MA, USA) at the Department of Inorganic and Analytical Chemistry, University of Debrecen. The concentration of the samples was 1 mg/mL and the solvent was water or acetonitrile. The instrument was equipped with an electrospray ion source, where the voltage was 4.5 kV. The drying gas was N_2_. The flow rate was 4 L/min and the drying temperature was 200 °C. Na-formate was injected after each measurement, enabling internal *m*/*z* calibration. The spectra were evaluated with Bruker Compass Data Analysis 4.4. software.

### 3.5. Crystal Structure Analysis

The slow evaporation of aqueous or ethanolic samples containing the appropriate [Co(4N)]^3+^ cation and PyPropHpH ligand afforded single crystals of [Co(tpa)(PyPropHp)]_2_(ClO_4_)_3_(OH)·6H_2_O (**5**) and [Co(tren)(PyPropHpH)](PF_6_)_2_(Cl)·EtOH·2H_2_O (**6**), respectively, suitable for X-ray diffraction studies. Analysis of **5** and **6** was carried out at the Physical Chemistry Department, University of Debrecen. A suitable crystal was fixed under a microscope onto a Mitegen loop using high-density oil. Diffraction intensity data collection was carried out using a Bruker-D8 Venture diffractometer (Bruker AXS GmbH, Karlsruhe, Germany) equipped with INCOATEC IμS 3.0 dual (Cu and Mo) sealed tube micro sources and Photon II Charge-Integrating Pixel Array detector (Bruker AXS GmbH, Karlsruhe, Germany) using Mo Kα (λ = 0.71073 Å) or Cu Kα (λ = 1.54178 Å) radiation. The structures were determined using RT data collection. High multiplicity data collection and integration was performed using the APEX3 (Ver. 2017.3–0, Bruker AXS Inc., Fitchburg, WI, USA, 2017) software. Data reduction and multi-scan absorption correction were performed using SAINT (Ver. 8.38A, Bruker AXS Inc., 2017). The structures could be solved using direct methods and refined on F2 using the SHELXL program incorporated into the APEX3 suite. Refinement was performed anisotropically for all non-hydrogen atoms. Hydrogen atoms were placed into geometric positions except N–H and O–H protons which were located on the difference electron density map and the N–H or O–H distances were constrained. The CIF file was merged and manually edited using the Publcif (version 1.9.22_c) software. The results of the X-ray diffraction structure determinations were good according to the Checkcif of PLATON software (Version 141123) and the structural parameters such as bond length and angle data were in the expected range. The location and orientation of solvent water molecules were not fully defined and the disordered PF_6_^–^ anion in the case of **5** resulted in several A and B level errors, but these are not relevant from the point of view of the coordination mode of the ligand in the cobalt complex. The solid state structures are stabilized by strong Coulombic interactions and hydrogen bonds. Further details of the X-ray studies are shown in the Supplementary Material (Table S2). The X-ray crystallographic data were deposited at the Cambridge Crystallographic Data Center with deposition numbers 2,375,424 and 2,375,425 for [Co(tpa)(PyPropHp)]_2_(ClO_4_)_3_(OH)·6(H_2_O) (**5**) and [Co(tren)(PyPropHpH)](PF_6_)_2_(Cl)·EtOH·2(H_2_O) (**6**), respectively. These data can be obtained free of charge via https://www.ccdc.cam.ac.uk/structures/ (accessed on 14 December 2024).

### 3.6. Cyclic Voltammetry

CV measurements were performed within the voltage range of 100 to −1200 mV, at RT in acetonitrile, using a BASi Epsilon Eclipse instrument (Tucson, AZ, USA) equipped with a three-electrode system consisting of an Ag/AgCl/3M KCl reference electrode (E_1/2_ = +209 mV vs. NHE), a platinum wire auxiliary electrode (ALS Co., Osaka, Japan) and a glassy carbon working electrode (CHI104). Aqueous solution of K_3_[Fe(CN)_6_] was used to calibrate the system (E_1/2_ = +458 mV vs. NHE in 0.50 M KCl). The samples were degassed before measurements using Ar gas. The concentration of the samples was 1–3 mM and the potential sweep rates were 100 mV/s during the determination of the redox potentials, and 0.10 M TBAF was used as the supporting electrolyte.

### 3.7. Stability Measurements

The stability of the complexes in PBS was monitored using UV–Vis measurement. The complexes were dissolved in PBS to a concentration of ~200 μM. The UV–Vis spectra of the resulting solutions at RT were recorded at different time points. The recording of spectra was done in the interval 250–600 nm using a Perkin Elmer Lambda 25 UV/Vis type spectrophotometer (Shelton, CT, USA).

### 3.8. Lipophilicity Measurements

The shake-flask method was used to measure the distribution coefficients (D) of the complexes in n-octanol/PBS pH = 7.40 at RT. PBS and n-octanol were mutually pre-saturated 1 week prior to use and separated. The complexes were dissolved in pre-saturated PBS solution to a concentration of ~100 μM. The resulting solutions were premixed with pre-saturated n-octanol for 72 h. The UV–Vis spectra of complexes in the aqueous phase were recorded. The aqueous solutions were mixed with pre-saturated n-octanol at a 1:10 volume ratio for 24 h. After phase separation, the UV–Vis spectra of the complexes in the aqueous phase were compared to that of the original solutions and the corresponding D values were calculated according to the following equation:

$$D=\left[\frac{A\left(aqueousphasebeforeseparation\right)}{A\left(aqueousphaseafterseparation\right)}-1\right]\times \frac{V\left(aqueousphase\right)}{V\left(octanolphase\right)}$$

The UV–Vis spectra were recorded in the interval 250–600 nm using a Perkin Elmer Lambda 25 UV/Vis type spectrophotometer. Phase separation was done using a Scanspeed 406 centrifuge (Labogene, Lynga, Denmark) at 4000 rpm for 5 min.

### 3.9. Cell Culture

Commercial-grade reagents and materials for biological work were used as received from commercial suppliers. The MCF-7 human breast adenocarcinoma cell line was purchased from ATCC (American Type Culture Collection). The cells were cultured in DMEM supplemented with L-glutamine, 10% FBS and 1% penicillin/streptomycin in a humidified chamber at 37 °C with 5% CO_2_ and subcultured twice a week with an appropriate plating density. During the treatment of compounds, the cells were incubated either in the same humidified chamber, denoted as normoxia, or in a hypoxic chamber at 37 °C with 5% CO_2_ and 1% O_2_, denoted as hypoxia.

### 3.10. Cytotoxicity Studies

Antiproliferative activities of the compounds were evaluated using an assay based on 3-(4,5-dimethylthiazol-2-yl)-2,5-diphenyltetrazolium bromide (MTT). Both under normoxia and hypoxia, MCF-7 cells were seeded and preincubated overnight, followed by 24, 48 or 72 h treatment with the substances at different concentrations. All treatment conditions were carried out in 6 replicates. After treatment, MTT was added to each well to a final concentration of 0.45 mg/mL and incubated for an additional 3 h. The medium was replaced by isopropyl alcohol to dissolve the formazan product. Absorbance was measured with a microplate reader (FLUOstar OPTIMA, BMG Labtech, Ortenberg, Germany) at 570 and 690 nm. The values were expressed relative to the untreated control And 10% DMSO was used as the positive control. At least 3 independent experiments were performed.

### 3.11. Gene Expression Analysis

The expression levels of TfR1, Ndrg1 and p21 were analyzed by qRT-PCR. Both under normoxia and hypoxia, MCF-7 cells were seeded and preincubated overnight, followed by 24 h treatment of PyPropHpH and **2** at 20 and 200 μM, or 72 h treatment of PyPropHpH and **2** at 100 μM. Total RNA was isolated using TRI reagent (Sigma-Aldrich). RNA from each sample (2000 ng) was reverse transcribed to cDNA using a Tetro cDNA Synthesis Kit in a final volume of 20 µL. PCR of cDNA was performed using the primer sets used in the literature [17]. HPRT1 was used as the internal reference gene. mRNA levels were measured using iQ™ SYBR^®^ Green Supermix (Bio-Rad Laboratories Inc., Hercules, CA, USA). Reactions were conducted according to the manufacturer’s protocol using the MyiQ2 two-color real-time PCR detection system (Bio-Rad Laboratories Inc.). All real-time amplifications were measured in triplicates. Results were evaluated with Bio-Rad iQ5 software (Bio-Rad Laboratories Inc.) and changes in mRNA levels were calculated using the 2^40-Ct^ method. The PCR protocol included an initial enzyme activation (95 °C for 3 min), then 40 cycles of amplification (95 °C for 15 s, 65 °C for 1 min), followed by a final annealing step (55 °C for 20.5 min). At least three independent experiments were performed.

## 4. Conclusions

Two Co(III) monometallic and two Co(III)/Ru(II) bimetallic complexes, differing in the tetradentate nitrogen ligands (tren or tpa), of PyPropHpH were synthesized and subjected to analytical and biological studies. The coordination of the (O,O) chelating set to the octahedral [Co(4N)]^3+^ core and the (N,N) donor to the half-sandwich Ru moiety was proven by ^1^H-NMR, ESI-MS and X-ray crystallographic data, consistent with the previous solution study [16]. Electrochemical results showed that the reduction potentials of the tpa complexes are less negative than those of the tren complexes, in agreement with our earlier findings on Co(4N) type complexes [22,23]. The cathodic peak potentials within −200 to −400 mV vs. NHE of the tpa complexes are low enough to be inert under normoxia and high enough to be reduced under hypoxia, as suggested by Hambley et al. [24,29,30]. Indeed, cytotoxicity studies on the MCF-7 cell line demonstrated hypoxia activation of the tpa complexes but not the tren complexes. All the complexes displayed low antiproliferative activity toward MCF-7 cells, probably owing to their relatively high hydrophilicity and low cellular uptake.

PyPropHpH was found to be moderately active under normoxia but inactive under hypoxia, further contributing to the low potency of the tpa complexes. Despite this, the induction of genes associated with iron depletion by PyPropHpH was observed under both normoxia and hypoxia. Existing literature about cancer cell resistance to iron chelators is scarce [31]; the relationship between drug resistance and hypoxia has been extensively investigated for clinically approved anticancer drugs but not for iron chelators, underscoring the need for further research on this topic [27,32]. Within the current work, detailed qRT-PCR studies of **2** showed that it acts as an inactive prodrug under normoxia and is bioreductively activated by releasing PyPropHpH after 72 h incubation under hypoxia.

In conclusion, our study has provided strong proof for the “activation by reduction” mechanism of a Co(III) prodrug system. Selective bioreduction was observed for complexes with ~−300 mV reduction potentials. However, complexes of PyPropHpH exhibited drawbacks, including their high hydrophilicity and the resistance of cancer cells to the iron-sequestering effect. Future design of the ambidentate ligands or 4N donor ligands needs to overcome these drawbacks while maintaining the appropriate reduction potentials of the complexes with the choice of the tetradentate 4N ligand.

## Figures and Tables

**Table 1:** Cathodic peak potential (E_pc_) values (mV vs. NHE) of the four complexes using glassy carbon electrode, c_complex_ = 1–3 mM in acetonitrile, I = 0.10 M TBAF, at a sweep rate of 100 mV/s.

Complex	Metal Centre	4N Ligand	E_pc_ (mV), Mean ± SD
**2**	Co	tpa	−298 ± 17
**4**	Co/Ru	tpa	−242 ± 17
**1**	Co	tren	−535 ± 19
**3**	Co/Ru	tren	−478 ± 18

**Table 2:** Distribution coefficient log D_n-octanol/PBS_ values determined for the four complexes at pH = 7.40, RT.

Complex	Metal Center	4N	log D_n-octanol/PBS_
**2**	Co	tpa	<–2.0
**4**	Co/Ru	tpa	–1.9
**1**	Co	tren	<–2.0
**3**	Co/Ru	tren	<–2.0

## Data Availability

Data are contained within the article and Supplementary Materials.

## Supplementary Materials

The following supporting information can be downloaded at: https://www.mdpi.com/article/10.3390/molecules29245967/s1, Figures S1–S6: ^1^H-NMR spectra of complexes **1**–**4**; Figure S7: Explanation of the likely isomers formed during the complexation of the heterobimetallic complexes; Figures S8 and S9: COSY spectrum of complex **3**; Figure S10: HR-ESI-MS spectra of the parent species of all complexes; Figure S11: Time-dependent UV–Vis spectra of the complexes **1**–**4**; Figures S12 and S13: Cell viability after 24 h and 48 h treatment of **2** and **4**; Table S1: List of identified species in HR-ESI-MS spectra; Table S2: X-ray crystallographic data of **5** and **6**. Reference [33] is cited in the Supplementary Materials.

## Abbreviations

ANOVA, analysis of variance; CDK, cyclin-dependent kinase; CV, cyclic voltammetry; DMEM, Dulbecco’s modified Eagle’s medium; ESI-MS, electrospray ionization mass spectrometry; FBS, fetal bovine serum; HIF-1, hypoxia-inducible factor 1; HPRT1, hypoxanthine phosphoribosyltransferase 1; IC50: 50% inhibitory concentration; Ndrg1, N-myc downstream regulated gene 1; NHE, normal hydrogen electrode; NMR, nuclear magnetic resonance; PBS, phosphate buffered saline; PGM, platinum group metal; p21, cyclin-dependent kinase inhibitor 1; ROS, reactive oxygen species; RT, room temperature; qRT-PCR, quantitative reverse transcription—polymerase chain reaction; SD, standard deviation; TfR1, transferrin receptor 1; UV-Vis, ultraviolet and visible; 95% CI, 95% confidence interval.
