# Supplementary material for: Synthesis and Characterization of Novel Co(III)/Ru(II) Heterobimetallic Complexes as Hypoxia-Activated Iron-Sequestering Anticancer Prodrugs
Source: Molecules. 2024 Dec 18;29(24):5967. doi: 10.3390/molecules29245967 (PMC11677873; doi:10.3390/molecules29245967)
Supplement: Supplementary file 1 [file molecules-29-05967-s001.zip › molecules-3362535-supplementary.pdf]

Synthesis and characterization of novel Co(III)/Ru(II) heterobimetallic complexes as hypoxia-activated iron-sequestering anticancer prodrugs

Tan Ba Tran [1], Éva Sipos [2], Attila Csaba Bényei [3], Sándor Nagy [1], István Lekli [2], Péter Buglyó [1,]*

[1] Department of Inorganic & Analytical Chemistry, Faculty of Science & Technology, University of Debrecen, H-4032 Debrecen, Hungary

[2] Department of Pharmacology, Faculty of Pharmacy, University of Debrecen, H-4032 Debrecen, Hungary

[3] Department of Physical Chemistry, Faculty of Science & Technology, University of Debrecen, H-4032 Debrecen, Hungary

Supporting information

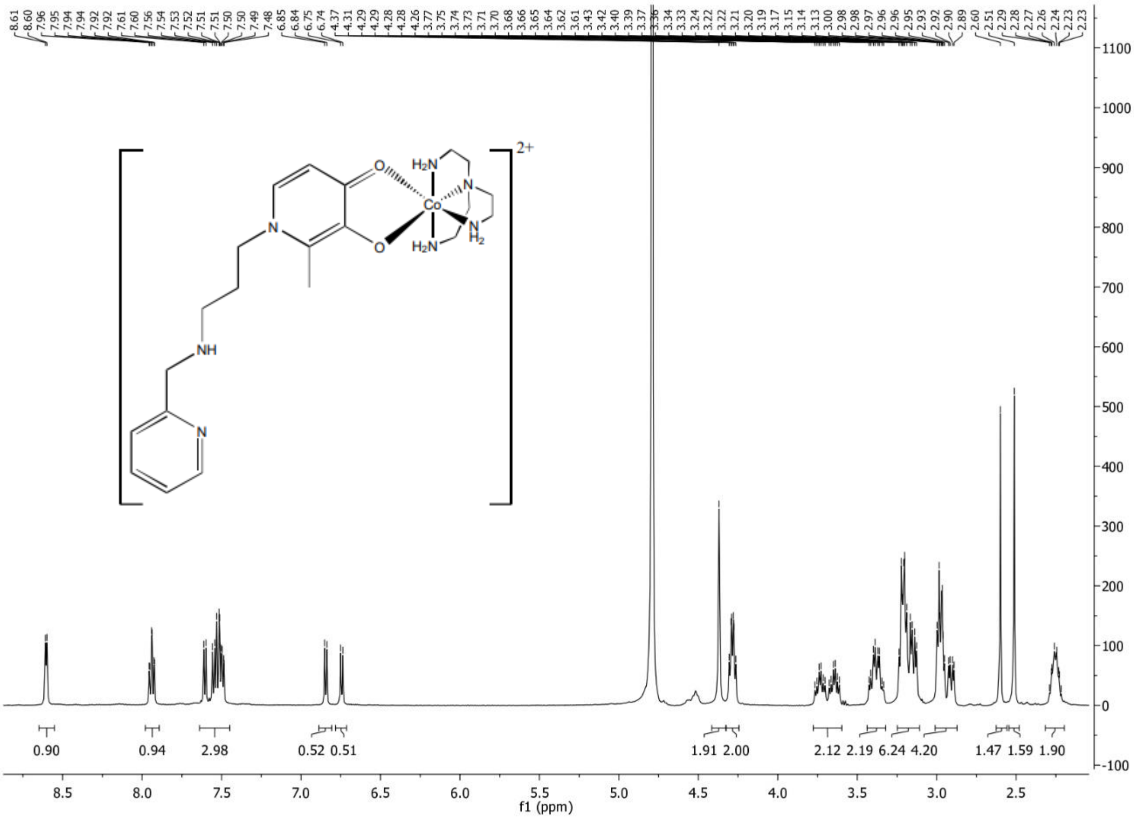

Figure S1. ^{1}H-NMR spectrum of **1** in D_2O.

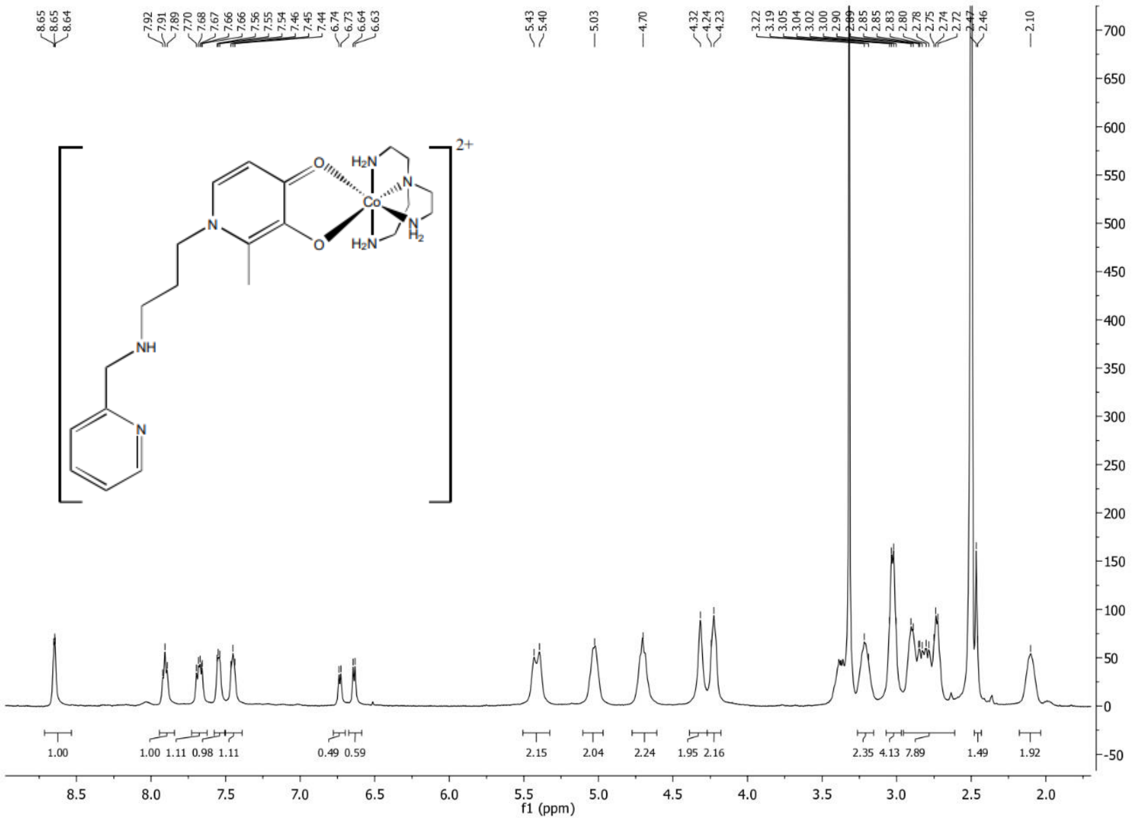

Figure S2. ^{1}H-NMR spectrum of **1** in DMSO-d^6.

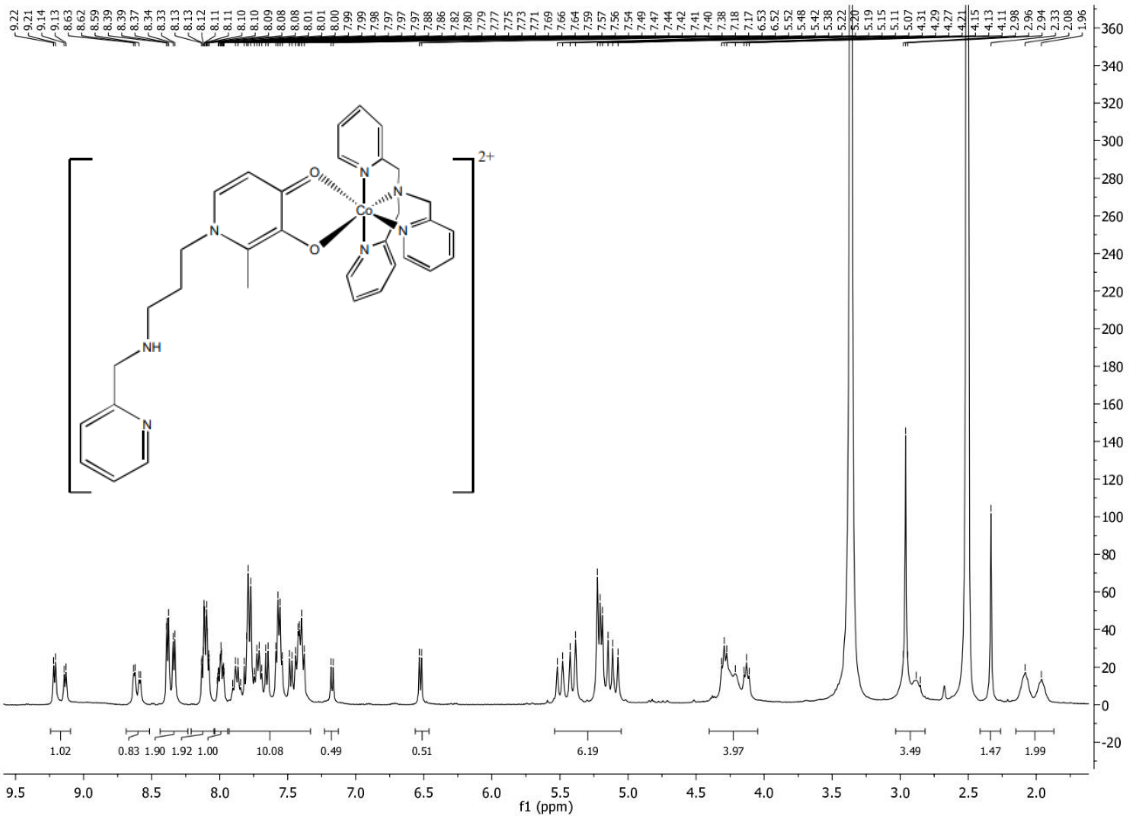

Figure S3. ^{1}H-NMR spectrum of **2** in DMSO-*d*^{6}.

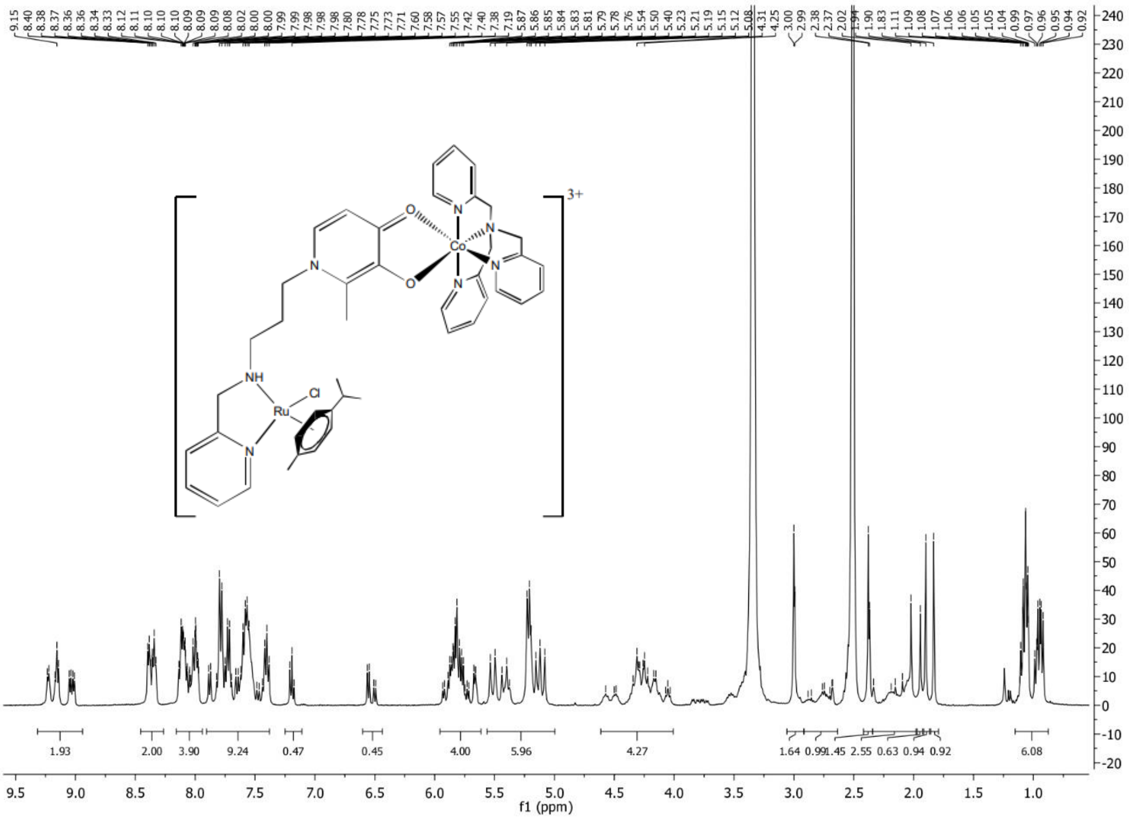

Figure S4. ^{1}H-NMR spectrum of **4** in DMSO-d^{6}.

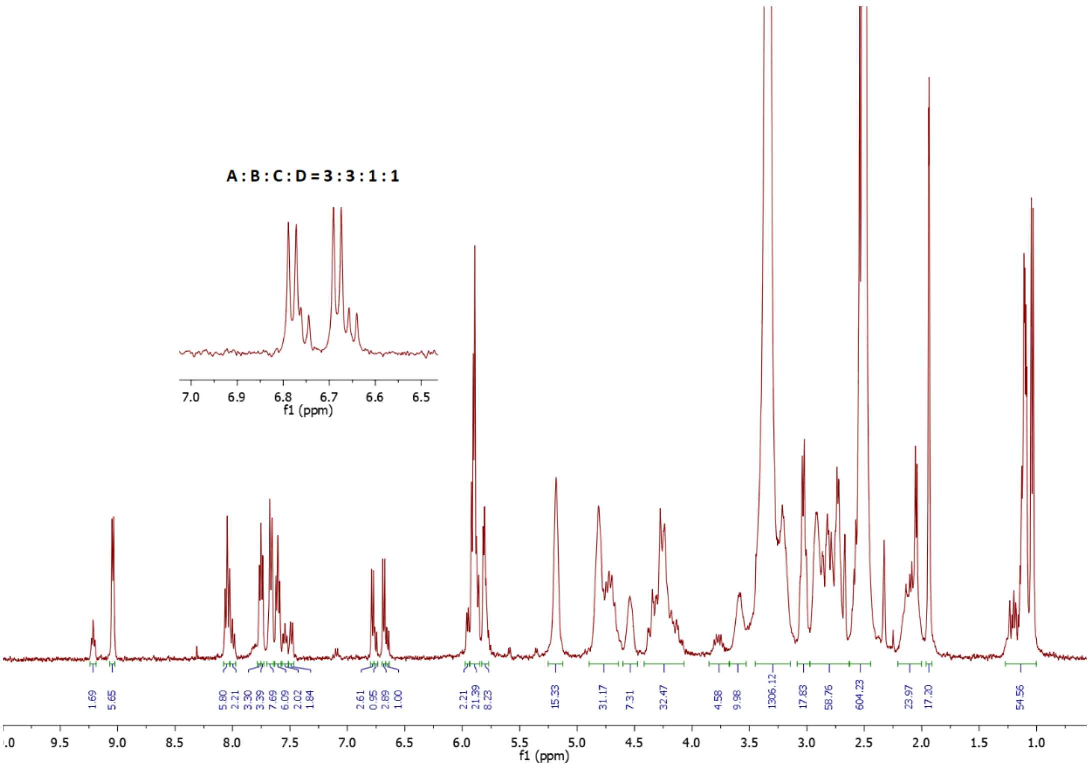

Figure S5. ^{1}H-NMR spectrum of **3** in DMSO-*d*[6]. Inlet: the four distinct signals of one of the pyridinone ring protons that belong to the isomers.

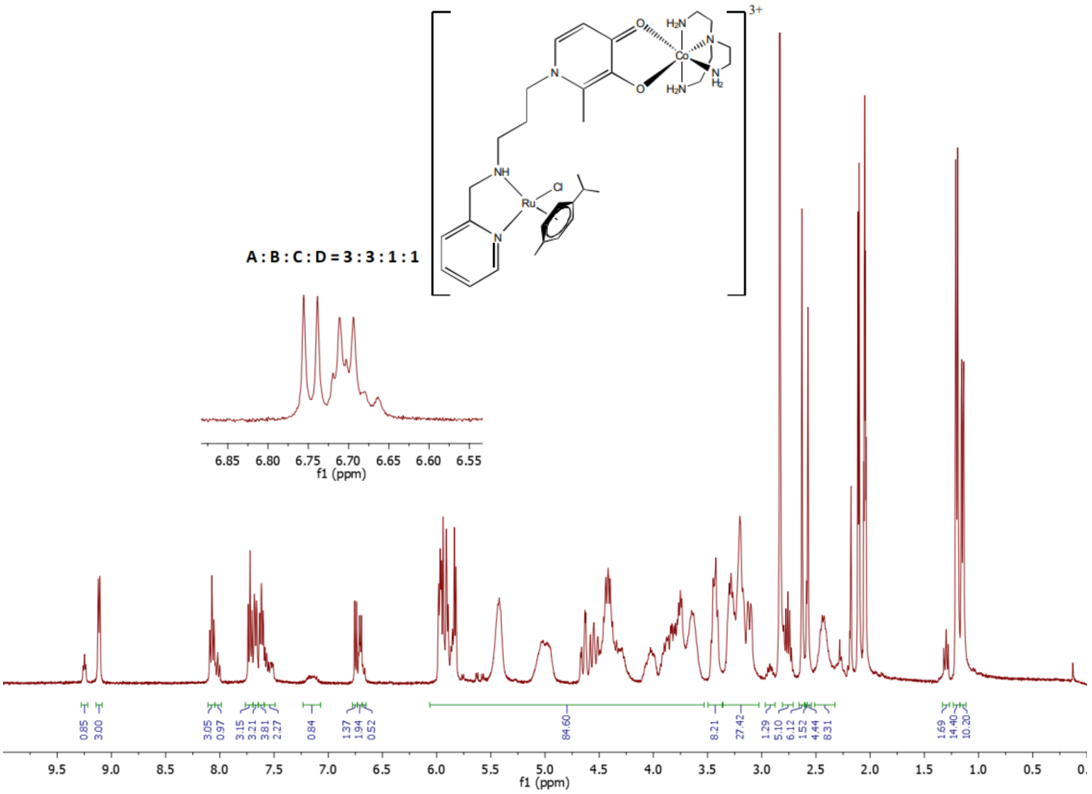

Figure S6. ^{1}H-NMR spectrum of **3** in acetone-d[6]. Inlet: the four distinct signals of one of the pyridinone ring protons that belong to the isomers.

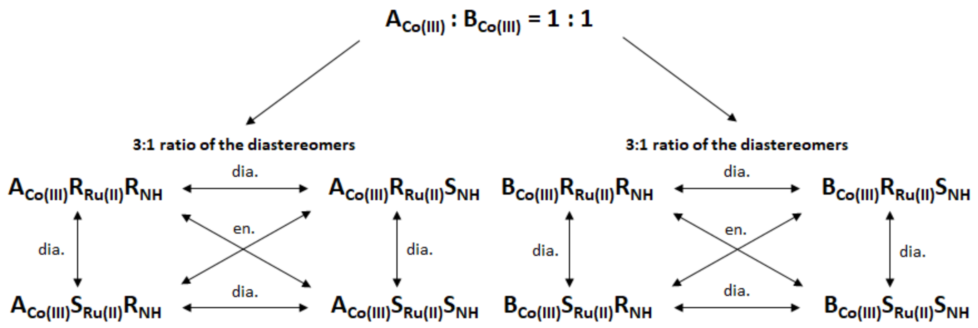

Figure S7. Explanation of the possible isomers formed during the complexation of the heterobimetallic complexes. The four isomers can be described as two diastereomers (dia.) at the Ru(II) metal centre (R and S), originating from the geometric isomers of the starting (O,O) coordinated Co(III) complexes (cis (A) and trans (B)) due to the asymmetric nature of the chelate formed. Coordination of the lone electron pair of the secondary NH group to the Ru(II) can create a new chiral centre, resulting in four enantiomers (en.) of the mentioned isomers, which cannot be detected by NMR measurements under these conditions.

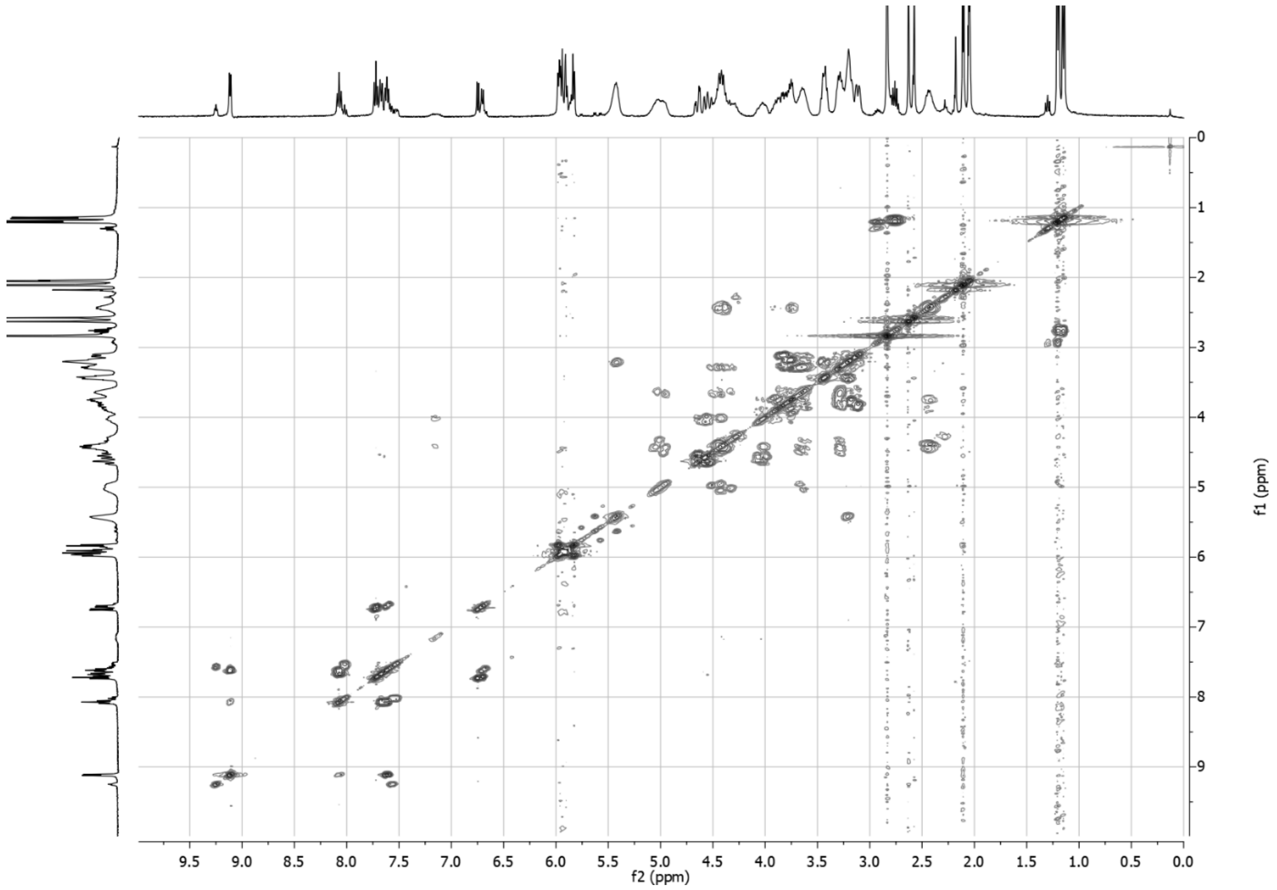

Figure S8. COSY spectrum of **3**.

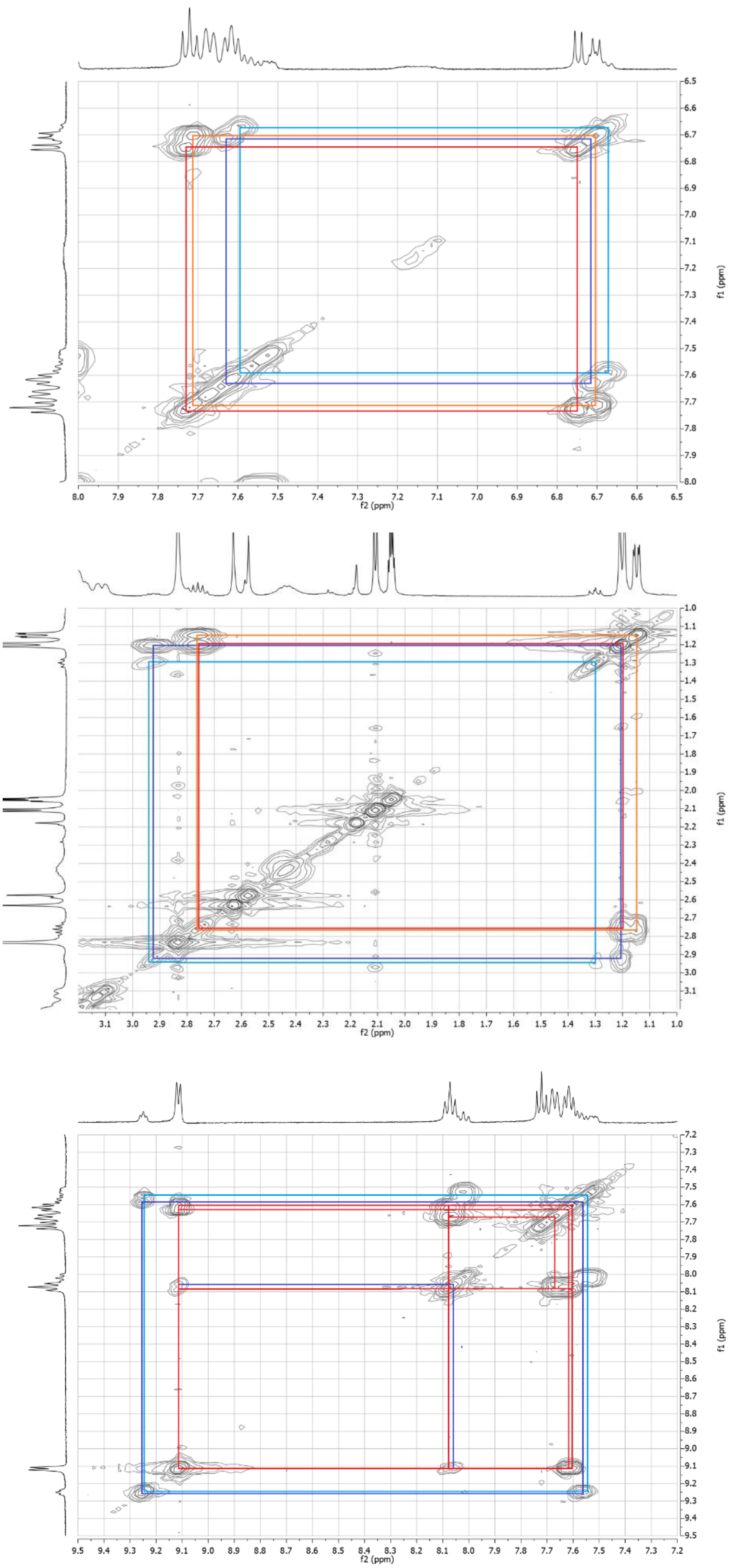

Figure S9. Spin systems of the isomers of the hydrogens of the various pyridinone (top), *p*-cymene (middle) and pyridine (bottom) moieties in **3.** Orange and red colours indicate the major while blue and navy blue the minor isomers.

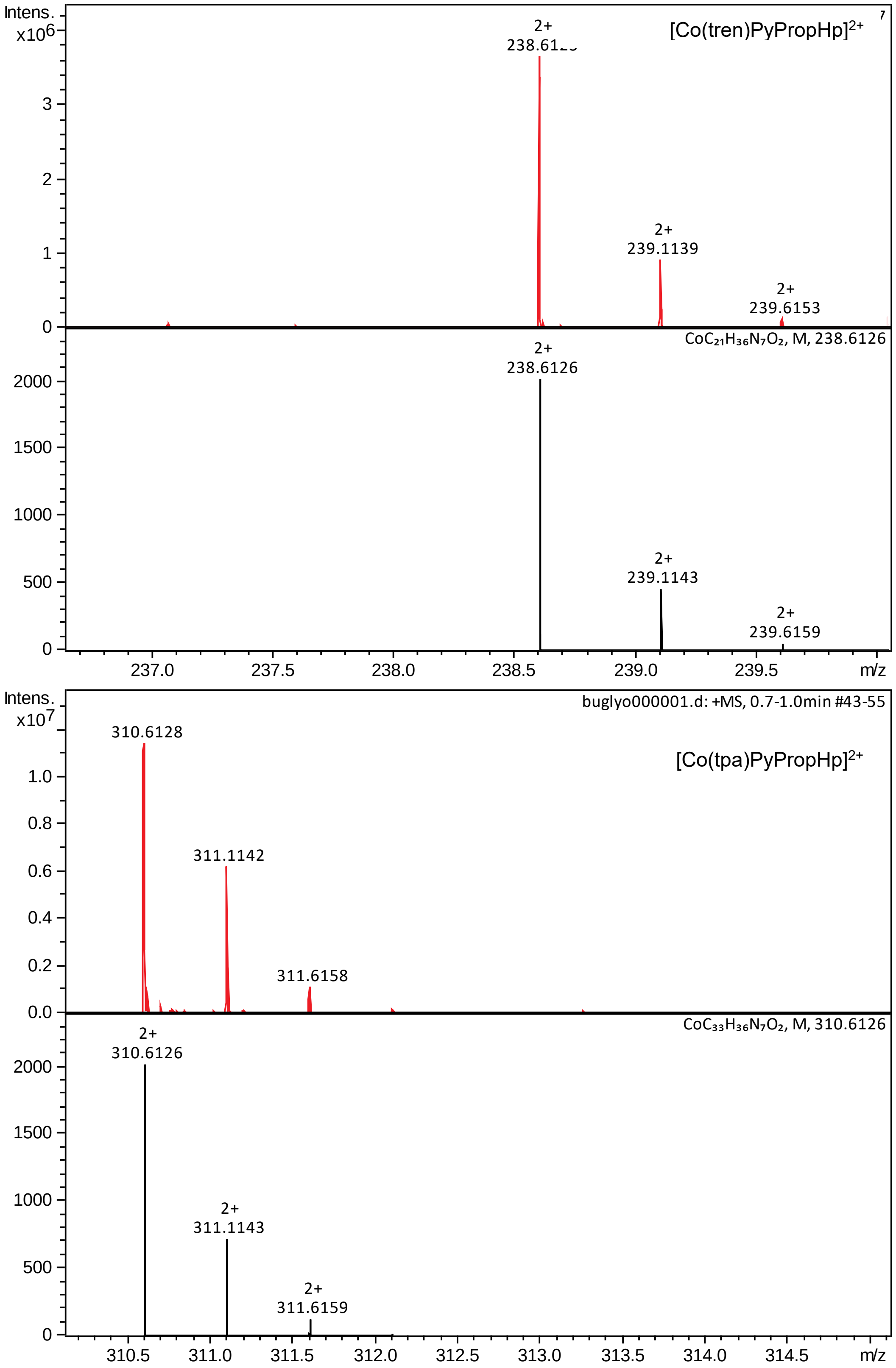

Figure S10. Measured (red) and calculated (black) isotope pattern of the major species of the various complexes.

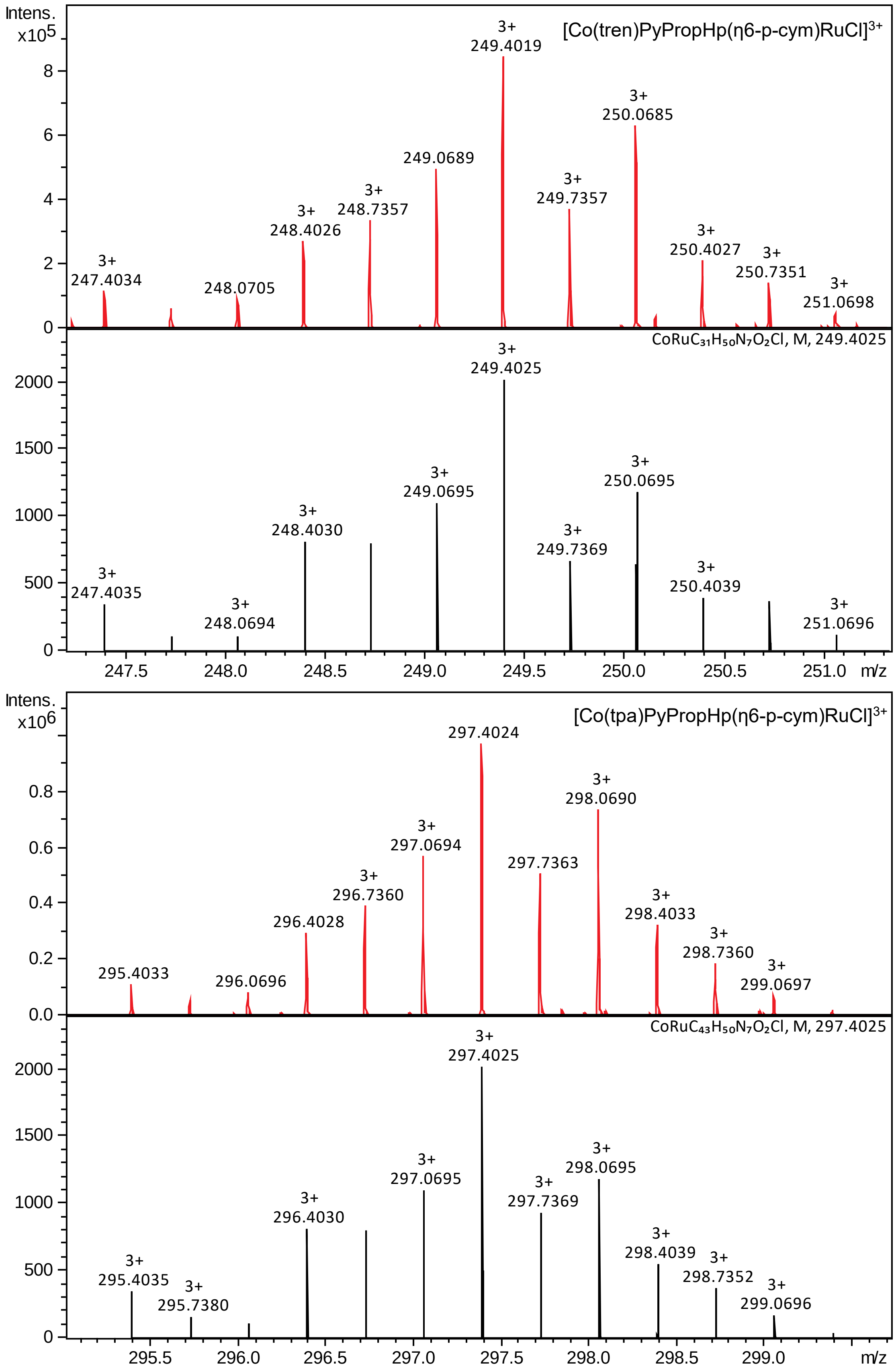

Figure S10 (cont.). Measured (red) and calculated (black) isotope pattern of the major species of the various complexes.

Table S1. Obtained and calculated *m/z* values for the identified species of the four complexes in their HRMS spectra.

**Sample**	**Identified species**	***m/z***	
		**Obtained**	**Calculated**
(**1**) in water	$[Co(tren)(PyPropHp)]^{2+}$	238.6123	238.6126
	$[Co(tren)H_{-1}(PyPropHp)]^{+}$	476.2177	476.2179
	$[PyPropHpH]^{+}$	273.1471	273.1472
(**2**) in acetonitrile	$[Co(tpa)(PyPropHp)]^{2+}$	310.6128	310.6126
	$[Co(tpa)OH_2]^{2+}$	183.5475	183.5479
	$[Co(tpa)]^{2+}$	174.5428	174.5426
(**3**) in acetonitrile	$[Co(tren)(PyPropHp)(\eta^6\text{-}p\text{-cym})RuCl]^{3+}$	249.4019	249.4025
	$[Co(tren)H_{-1}(PyPropHp)(\eta^6\text{-}p\text{-cym})Ru]^{3+}$	237.4097	237.4102
(**4**) in acetonitrile	$[Co(tpa)(PyPropHp)(\eta^6\text{-}p\text{-cym})RuCl]^{3+}$	297.4024	297.4025
	$[Co(tpa)H_{-1}(PyPropHp)(\eta^6\text{-}p\text{-cym})Ru]^{3+}$	285.4100	285.4102
	$[Co(tpa)(PyPropHp)]^{2+}$	310.6121	310.6126
	$[(\eta^6\text{-}p\text{-cym})Ru(PyPropHp)Cl]^{+}$	543.1224	543.1221
	$[Co(tpa)OH_2]^{2+}$	183.5474	183.5479
	$[Co(tpa)]^{2+}$	174.5427	174.5426

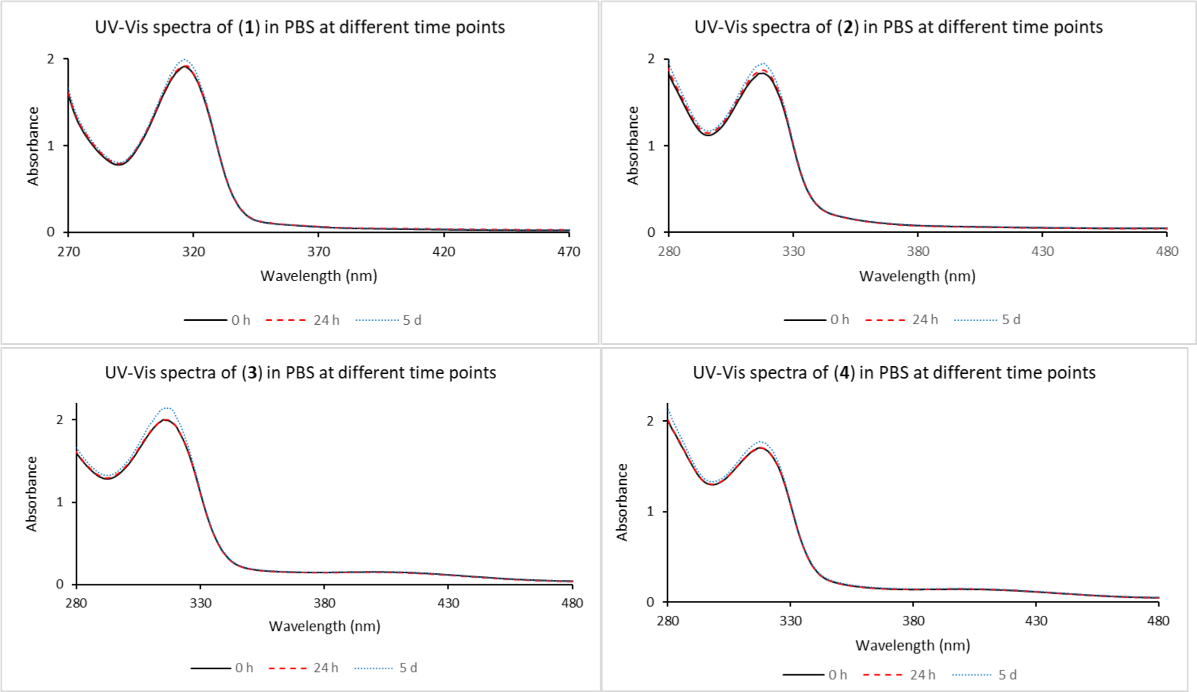

Figure S11. UV-Vis spectra of all complexes in PBS over time.

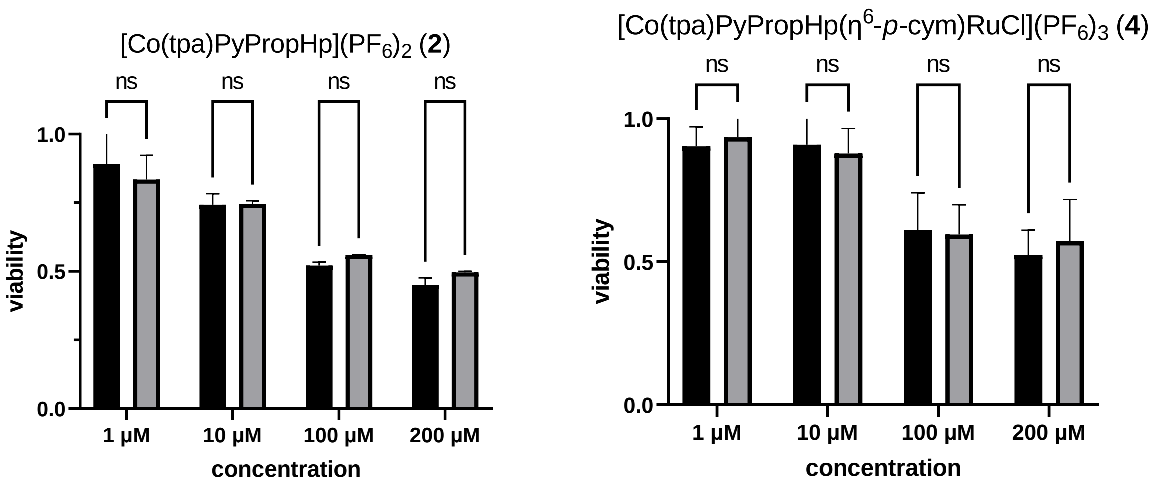

Figure S12. Cell viability after 24-hour treatment with **2** and **4** under normoxia (black bar) and hypoxia (gray bar). Data points are presented as mean (SD). Multiple paired t test and Holm-Šidák post-hoc test were used to analyse the data. Significance level: ns: $p > 0.05$, *: $p \leq 0.05$, **: $p \leq 0.01$, ***: $p \leq 0.001$, ****: $p \leq 0.0001$.

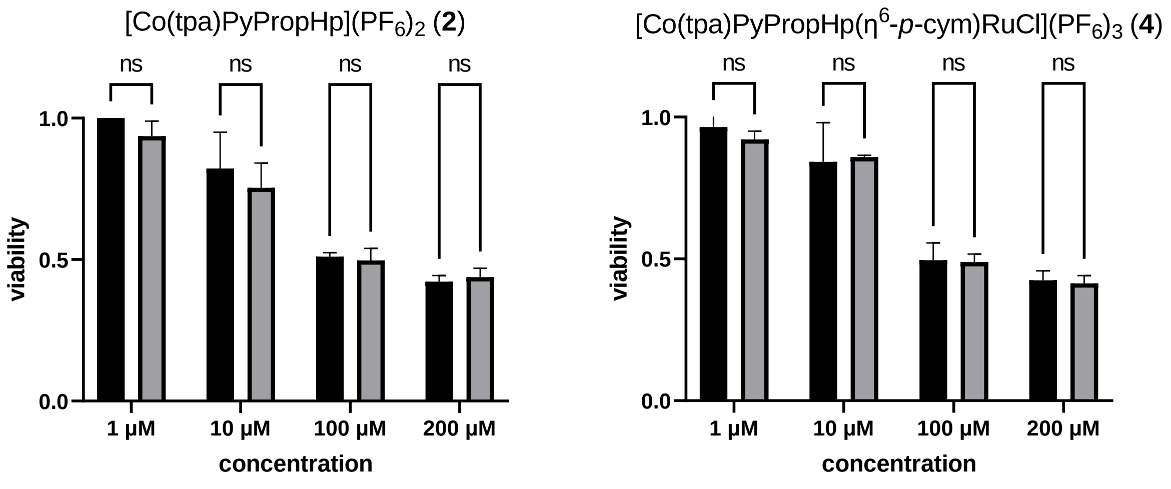

Figure S13. Cell viability after 48-hour treatment with **2** and **4** under normoxia (black bar) and hypoxia (gray bar). Data points are presented as mean (SD). Multiple paired t test and Holm-Šidák post-hoc test were used to analyse the data. Significance level: ns: $p > 0.05$, *: $p \leq 0.05$, **: $p \leq 0.01$, ***: $p \leq 0.001$, ****: $p \leq 0.0001$.

Table S2. Experimental details of X-ray diffraction studies.

	Co_tren_PyPropHpH	Co_tpa_PyPropHp
Crystal data		
Chemical formula	$C_{21}H_{37}CoN_7O_2 \cdot 2(F_6P) \cdot C_2H_6O \cdot 2(H_2O) \cdot Cl$	$2(C_{33}H_{36}CoN_7O_2) \cdot 3(ClO_4) \cdot HO \cdot 6(H_2O)$
M_r	1771.99	1666.68
Crystal system, space group	Triclinic, $P\bar{1}$	Orthorhombic, *Fdd*2
Temperature (K)	199	295
a, *b*, *c* (Å)	8.6024 (7), 14.0521 (10), 15.375 (1)	39.0638 (15), 45.1846 (16), 8.6277 (3)
α, β, γ (°)	66.058 (2), 83.948 (2), 77.511 (3)	90, 90, 90
V (Å^3)	1658.1 (2)	15228.6 (10)
Z	1	8
Radiation type	Mo $K\alpha$	
μ (mm^{-1})	0.81	0.63
Crystal size (mm)	0.29 × 0.22 × 0.08	0.26 × 0.23 × 0.07
Data collection		
Diffractometer	Bruker D8 VENTURE	
Absorption correction	Numerical *SADABS2016*/2 - Bruker AXS area detector scaling and absorption correction	Multi-scan *SADABS2016*/2 - Bruker AXS area detector scaling and absorption correction
T_{min}, T_{max}	0.67, 0.75	0.78, 0.96
No. of measured, independent and observed [$I > 2\sigma(I)$] reflections	38677, 6306, 4538	76148, 7255, 6310
R_{int}	0.091	0.113
$(\sin \theta/\lambda)_{max}$ (Å^{-1})	0.611	0.611
Refinement		
$R[F^2 > 2\sigma(F^2)]$, $wR(F^2)$, S	0.105, 0.294, 1.72	0.098, 0.219, 1.19
No. of reflections	6306	7255
No. of parameters	508	508
No. of restraints	69	10
H-atom treatment	H atoms treated by a mixture of independent and constrained refinement	
	$w = 1/[\sigma^2(F_o^2) + (0.1022P)^2 + 1.2311P]$ where $P = (F_o^2 + 2F_c^2)/3$	$w = 1/[\sigma^2(F_o^2) + (0.0974P)^2 + 83.5571P]$ where $P = (F_o^2 + 2F_c^2)/3$
$(\Delta/\sigma)_{max}$	6.140	0.930
$\Delta\rangle_{max}$, $\Delta\rangle_{min}$ (e Å^{-3})	2.14, -1.00	0.79, -0.85

Geometric parameters (Å, º) for (Co_tren_PyPropHpH)

C2—N1	1.346 (8)	C55—H55A	0.99
C2—C3	1.399 (8)	C55—H55B	0.99
C2—C20	1.500 (10)	C56—N57	1.542 (13)
C3—O3	1.331 (7)	C56—H56A	0.99
C3—C4	1.412 (9)	C56—H56B	0.99
C4—O4	1.304 (7)	C56—H57B	1.37 (9)
C4—C5	1.393 (8)	C58—N52	1.487 (8)
C5—C6	1.341 (9)	C58—C59	1.520 (10)
C5—H5	0.95	C58—H58A	0.99
C6—N1	1.392 (9)	C58—H58B	0.99
C6—H6	0.95	C59—N60	1.471 (9)
C11—N1	1.473 (7)	C59—H59A	0.99
C11—C12	1.518 (10)	C59—H59B	0.99
C11—H11A	0.99	O1W—O2W	1.71 (3)
C11—H11B	0.99	O1W—H1W	0.874 (10)
C12—C13	1.517 (9)	O1W—H2W	0.868 (11)
C12—H12A	0.99	O3—Co1	1.895 (4)
C12—H12B	0.99	O2W—H2W	1.44 (10)
C13—N14	1.474 (9)	O2W—H3W	0.928 (19)
C13—H13A	0.99	O2W—H4W	0.944 (15)
C13—H13B	0.99	O4—Co1	1.924 (4)
C20—H20A	0.98	O41—H41	1.00 (5)
C20—H20B	0.98	Co1—N57	1.938 (5)
C20—H20C	0.98	Co1—N52	1.939 (5)
C22—N21	1.321 (9)	Co1—N54	1.945 (6)
C22—C23	1.384 (10)	Co1—N60	1.955 (6)
C22—C27	1.507 (9)	F11—F12	1.57 (3)
C23—C24	1.383 (12)	F11—P1	1.709 (9)
C23—H23	0.95	F12—F16	1.45 (3)
C24—C25	1.356 (12)	F12—P1	1.710 (10)
C24—H24	0.95	F13—P1	1.694 (10)
C25—C26	1.354 (11)	F14—P1	1.705 (10)
C25—H25	0.95	F15—P1	1.694 (10)
C26—N21	1.361 (9)	F16—P1	1.680 (9)
C26—H26	0.95	F21—P2	1.399 (18)
C27—N14	1.483 (9)	F21—F22	1.61 (2)
C27—H27A	0.99	F22—P2	1.268 (12)
C27—H27B	0.99	F22—F23^{i}	1.78 (3)
C40—O41	1.15 (4)	F23—P2	1.556 (18)

C40—C41	1.54 (4)	F31—F32^{ii}	1.53 (3)
C40—H40A	0.99	F31—P3	1.708 (10)
C40—H40B	0.99	F32—P3^{ii}	0.59 (3)
C41—H41A	0.98	F32—F32^{ii}	1.20 (4)
C41—H41B	0.98	F32—P3	1.735 (10)
C41—H41C	0.98	F33—P3	1.741 (10)
C52—N52	1.490 (9)	P3—P3^{ii}	2.30 (3)
C52—C53	1.512 (11)	N14—H14A	0.860 (10)
C52—H52A	0.99	N14—H14B	0.860 (10)
C52—H52B	0.99	N54—H54A	0.862 (10)
C53—N54	1.504 (10)	N54—H54B	0.860 (10)
C53—H53A	0.99	N57—H57A	0.857 (10)
C53—H53B	0.99	N57—H57B	0.865 (10)
C55—C56	1.470 (13)	N60—H60A	0.862 (10)
C55—N52	1.491 (8)	N60—H60B	0.861 (10)
N1—C2—C3	119.2 (6)	C3—O3—Co1	108.3 (4)
N1—C2—C20	120.9 (6)	O1W—O2W—H2W	30.5 (8)
C3—C2—C20	119.8 (6)	O1W—O2W—H3W	82 (2)
O3—C3—C2	121.7 (6)	H2W—O2W—H3W	101 (5)
O3—C3—C4	118.2 (5)	O1W—O2W—H4W	158 (2)
C2—C3—C4	120.1 (5)	H2W—O2W—H4W	133 (5)
O4—C4—C5	124.3 (6)	H3W—O2W—H4W	120 (3)
O4—C4—C3	117.1 (5)	C4—O4—Co1	109.1 (4)
C5—C4—C3	118.6 (5)	C40—O41—H41	137 (8)
C6—C5—C4	119.8 (6)	O3—Co1—O4	87.21 (17)
C6—C5—H5	120.1	O3—Co1—N57	92.5 (2)
C4—C5—H5	120.1	O4—Co1—N57	178.9 (2)
C5—C6—N1	121.7 (6)	O3—Co1—N52	179.6 (2)
C5—C6—H6	119.2	O4—Co1—N52	93.04 (19)
N1—C6—H6	119.2	N57—Co1—N52	87.3 (2)
N1—C11—C12	110.5 (5)	O3—Co1—N54	93.0 (2)
N1—C11—H11A	109.6	O4—Co1—N54	86.4 (2)
C12—C11—H11A	109.6	N57—Co1—N54	92.6 (3)
N1—C11—H11B	109.5	N52—Co1—N54	87.3 (2)
C12—C11—H11B	109.5	O3—Co1—N60	93.1 (2)
H11A—C11—H11B	108.1	O4—Co1—N60	87.8 (2)
C13—C12—C11	110.5 (6)	N57—Co1—N60	93.3 (3)
C13—C12—H12A	109.5	N52—Co1—N60	86.5 (2)
C11—C12—H12A	109.5	N54—Co1—N60	171.3 (3)
C13—C12—H12B	109.6	F12—F11—P1	62.8 (6)

C11—C12—H12B	109.6	F16—F12—F11	118.5 (14)
H12A—C12—H12B	108.1	F16—F12—P1	63.5 (7)
N14—C13—C12	111.3 (6)	F11—F12—P1	62.7 (8)
N14—C13—H13A	109.3	F12—F16—P1	65.7 (7)
C12—C13—H13A	109.3	P2—F21—F22	49.2 (8)
N14—C13—H13B	109.4	P2—F22—F21	56.7 (8)
C12—C13—H13B	109.4	P2—F22—F23^{i}	58.5 (9)
H13A—C13—H13B	108.0	F21—F22—F23^{i}	82.6 (13)
C2—C20—H20A	109.5	P2—F23—F22^{i}	44.0 (7)
C2—C20—H20B	109.5	F32^{ii}—F31—P3	20.0 (11)
H20A—C20—H20B	109.5	P3^{ii}—F32—F32^{ii}	149 (5)
C2—C20—H20C	109.5	P3^{ii}—F32—F31^{ii}	98 (3)
H20A—C20—H20C	109.5	F32^{ii}—F32—F31^{ii}	94 (3)
H20B—C20—H20C	109.5	P3^{ii}—F32—P3	159 (3)
N21—C22—C23	124.2 (7)	F32^{ii}—F32—P3	10.2 (15)
N21—C22—C27	118.1 (6)	F31^{ii}—F32—P3	89.9 (15)
C23—C22—C27	117.6 (7)	F16—P1—F13	91.4 (13)
C24—C23—C22	117.9 (8)	F16—P1—F15	138.1 (15)
C24—C23—H23	121.0	F13—P1—F15	127.8 (18)
C22—C23—H23	121.1	F16—P1—F14	66.7 (7)
C25—C24—C23	118.8 (8)	F13—P1—F14	146.4 (19)
C25—C24—H24	120.6	F15—P1—F14	71.6 (13)
C23—C24—H24	120.6	F16—P1—F12	50.8 (10)
C26—C25—C24	119.7 (8)	F13—P1—F12	65.5 (16)
C26—C25—H25	120.1	F15—P1—F12	127.0 (17)
C24—C25—H25	120.2	F14—P1—F12	81.0 (12)
C25—C26—N21	123.5 (8)	F16—P1—F11	100.0 (10)
C25—C26—H26	118.2	F13—P1—F11	81.3 (17)
N21—C26—H26	118.2	F15—P1—F11	75.6 (14)
N14—C27—C22	113.0 (6)	F14—P1—F11	78.0 (7)
N14—C27—H27A	109.0	F12—P1—F11	54.5 (11)
C22—C27—H27A	109.0	F22^{i}—P2—F22	180 (2)
N14—C27—H27B	109.0	F22^{i}—P2—F21^{i}	74.1 (10)
C22—C27—H27B	109.0	F22—P2—F21^{i}	105.9 (10)
H27A—C27—H27B	107.8	F22^{i}—P2—F21	105.9 (10)
O41—C40—C41	113 (3)	F22—P2—F21	74.1 (10)
O41—C40—H40A	110.3	F21^{i}—P2—F21	180.0
C41—C40—H40A	110.1	F22^{i}—P2—F23^{i}	102.5 (11)
O41—C40—H40B	106.8	F22—P2—F23^{i}	77.5 (11)
C41—C40—H40B	108.7	F21^{i}—P2—F23^{i}	81.4 (12)
H40A—C40—H40B	107.8	F21—P2—F23^{i}	98.6 (12)

C40—C41—H41A	110.4	F22^{i}—P2—F23	77.5 (11)
C40—C41—H41B	108.6	F22—P2—F23	102.5 (11)
H41A—C41—H41B	109.5	F21^{i}—P2—F23	98.6 (12)
C40—C41—H41C	109.4	F21—P2—F23	81.4 (12)
H41A—C41—H41C	109.5	F23^{i}—P2—F23	180.0
H41B—C41—H41C	109.5	F32^{ii}—P3—F31	62 (3)
N52—C52—C53	108.2 (6)	F32^{ii}—P3—F32	21 (3)
N52—C52—H52A	110.1	F31—P3—F32	71.0 (9)
C53—C52—H52A	110.0	F32^{ii}—P3—F33	89 (3)
N52—C52—H52B	110.1	F31—P3—F33	78.2 (8)
C53—C52—H52B	110.1	F32—P3—F33	71.4 (9)
H52A—C52—H52B	108.4	F32^{ii}—P3—P3^{ii}	16 (3)
N54—C53—C52	108.7 (6)	F31—P3—P3^{ii}	68.6 (10)
N54—C53—H53A	109.9	F32—P3—P3^{ii}	5.3 (8)
C52—C53—H53A	109.9	F33—P3—P3^{ii}	75.8 (9)
N54—C53—H53B	110.0	C2—N1—C6	120.5 (5)
C52—C53—H53B	110.0	C2—N1—C11	123.4 (6)
H53A—C53—H53B	108.3	C6—N1—C11	115.9 (5)
C56—C55—N52	111.8 (6)	C13—N14—C27	114.5 (6)
C56—C55—H55A	109.2	C13—N14—H14A	93 (6)
N52—C55—H55A	109.3	C27—N14—H14A	101 (6)
C56—C55—H55B	109.2	C13—N14—H14B	107 (6)
N52—C55—H55B	109.2	C27—N14—H14B	114 (6)
H55A—C55—H55B	107.9	H14A—N14—H14B	126 (9)
C55—C56—N57	108.0 (8)	C22—N21—C26	115.7 (6)
C55—C56—H56A	110.1	C58—N52—C52	110.7 (5)
N57—C56—H56A	110.1	C58—N52—C55	111.9 (5)
C55—C56—H56B	110.1	C52—N52—C55	111.7 (6)
N57—C56—H56B	110.1	C58—N52—Co1	105.7 (4)
H56A—C56—H56B	108.4	C52—N52—Co1	105.7 (4)
C55—C56—H57B	95.7 (17)	C55—N52—Co1	110.8 (4)
N57—C56—H57B	33.9 (6)	C53—N54—Co1	110.1 (4)
H56A—C56—H57B	142.8	C53—N54—H54A	98 (6)
H56B—C56—H57B	86.1	Co1—N54—H54A	116 (6)
N52—C58—C59	107.5 (6)	C53—N54—H54B	123 (6)
N52—C58—H58A	110.2	Co1—N54—H54B	108 (6)
C59—C58—H58A	110.2	H54A—N54—H54B	102 (7)
N52—C58—H58B	110.2	C56—N57—Co1	108.2 (5)
C59—C58—H58B	110.2	C56—N57—H57A	99 (6)
H58A—C58—H58B	108.5	Co1—N57—H57A	111 (5)
N60—C59—C58	108.0 (5)	C56—N57—H57B	62 (6)

N60—C59—H59A	110.1	Co1—N57—H57B	116 (2)
C58—C59—H59A	110.1	H57A—N57—H57B	133 (6)
N60—C59—H59B	110.1	C59—N60—Co1	110.7 (5)
C58—C59—H59B	110.1	C59—N60—H60A	106 (5)
H59A—C59—H59B	108.4	Co1—N60—H60A	123 (6)
O2W—O1W—H1W	111 (8)	C59—N60—H60B	103 (5)
O2W—O1W—H2W	57 (7)	Co1—N60—H60B	105 (5)
H1W—O1W—H2W	110 (10)	H60A—N60—H60B	107 (8)
N1—C2—C3—O3	179.8 (6)	F21—F22—P2—F21^{i}	180.002 (3)
C20—C2—C3—O3	-0.9 (10)	F23^{i}—F22—P2—F21^{i}	77.1 (14)
N1—C2—C3—C4	0.7 (10)	F23^{i}—F22—P2—F21	-102.9 (14)
C20—C2—C3—C4	180.0 (7)	F21—F22—P2—F23^{i}	102.9 (14)
O3—C3—C4—O4	0.2 (9)	F21—F22—P2—F23	-77.1 (14)
C2—C3—C4—O4	179.4 (6)	F23^{i}—F22—P2—F23	179.999 (2)
O3—C3—C4—C5	-179.4 (6)	F22—F21—P2—F22^{i}	180.000 (3)
C2—C3—C4—C5	-0.2 (10)	F22—F21—P2—F23^{i}	-74.3 (13)
O4—C4—C5—C6	-179.8 (7)	F22—F21—P2—F23	105.7 (13)
C3—C4—C5—C6	-0.2 (10)	F22^{i}—F23—P2—F22	180.000 (2)
C4—C5—C6—N1	0.2 (11)	F22^{i}—F23—P2—F21^{i}	-71.5 (12)
N1—C11—C12—C13	-173.7 (6)	F22^{i}—F23—P2—F21	108.5 (12)
C11—C12—C13—N14	-179.5 (6)	F32^{ii}—F31—P3—F32	-21 (4)
N21—C22—C23—C24	-1.3 (11)	F32^{ii}—F31—P3—F33	-95 (4)
C27—C22—C23—C24	175.1 (7)	F32^{ii}—F31—P3—P3^{ii}	-16 (3)
C22—C23—C24—C25	2.6 (12)	P3^{ii}—F32—P3—F32^{ii}	0.01 (2)
C23—C24—C25—C26	-0.9 (13)	F31^{ii}—F32—P3—F32^{ii}	111 (10)
C24—C25—C26—N21	-2.4 (13)	P3^{ii}—F32—P3—F31	62 (9)
N21—C22—C27—N14	-32.4 (9)	F32^{ii}—F32—P3—F31	62 (9)
C23—C22—C27—N14	151.0 (7)	F31^{ii}—F32—P3—F31	173.0 (12)
N52—C52—C53—N54	-44.2 (8)	P3^{ii}—F32—P3—F33	145 (10)
N52—C55—C56—N57	38.8 (12)	F32^{ii}—F32—P3—F33	145 (10)
N52—C58—C59—N60	47.0 (7)	F31^{ii}—F32—P3—F33	-103.5 (14)
C2—C3—O3—Co1	-177.1 (5)	F32^{ii}—F32—P3—P3^{ii}	-0.01 (6)
C4—C3—O3—Co1	2.0 (7)	F31^{ii}—F32—P3—P3^{ii}	111 (10)
C5—C4—O4—Co1	177.2 (6)	C3—C2—N1—C6	-0.6 (10)
C3—C4—O4—Co1	-2.4 (7)	C20—C2—N1—C6	-179.9 (7)
C3—O3—Co1—O4	-2.6 (4)	C3—C2—N1—C11	-177.1 (6)
C3—O3—Co1—N57	176.4 (4)	C20—C2—N1—C11	3.6 (11)
C3—O3—Co1—N54	83.7 (4)	C5—C6—N1—C2	0.2 (11)
C3—O3—Co1—N60	-90.2 (4)	C5—C6—N1—C11	176.9 (7)
P1—F11—F12—F16	-31.7 (17)	C12—C11—N1—C2	92.0 (8)

F11—F12—F16—P1	31.4 (16)	C12—C11—N1—C6	-84.6 (7)
P2—F21—F22—F23^{i}	56.9 (9)	C12—C13—N14—C27	-165.2 (6)
F12—F16—P1—F13	56 (2)	C22—C27—N14—C13	-65.8 (8)
F12—F16—P1—F15	-105 (3)	C23—C22—N21—C26	-1.8 (10)
F12—F16—P1—F14	-97.5 (15)	C27—C22—N21—C26	-178.1 (6)
F12—F16—P1—F11	-25.2 (16)	C25—C26—N21—C22	3.7 (11)
F11—F12—P1—F16	-148.9 (19)	C59—C58—N52—C52	-161.6 (5)
F16—F12—P1—F13	-114.1 (19)	C59—C58—N52—C55	73.1 (7)
F11—F12—P1—F13	97.0 (18)	C59—C58—N52—Co1	-47.6 (5)
F16—F12—P1—F15	126 (2)	C53—C52—N52—C58	160.7 (5)
F11—F12—P1—F15	-23 (2)	C53—C52—N52—C55	-73.9 (7)
F16—F12—P1—F14	67.2 (11)	C53—C52—N52—Co1	46.7 (6)
F11—F12—P1—F14	-81.7 (11)	C56—C55—N52—C58	-139.9 (9)
F16—F12—P1—F11	148.9 (19)	C56—C55—N52—C52	95.3 (10)
F12—F11—P1—F16	23.9 (15)	C56—C55—N52—Co1	-22.3 (10)
F12—F11—P1—F13	-66.0 (14)	C52—C53—N54—Co1	20.5 (8)
F12—F11—P1—F15	161.2 (19)	C55—C56—N57—Co1	-37.6 (11)
F12—F11—P1—F14	87.4 (15)	C58—C59—N60—Co1	-23.9 (7)

Symmetry codes: (i) -*x*, -*y*+1, -*z*; (ii) -*x*, -*y*+1, -*z*+1.

Hydrogen-bond geometry (Å, º) for (Co_tren_PyPropHpH)

D—H···*A*	*D*—H	H···*A*	*D*···*A*	*D*—H···*A*
C5—H5···O4^{iii}	0.95	2.64	3.591 (8)	178
C11—H11*A*···F11^{iv}	0.99	2.55	3.358 (13)	139
C11—H11*B*···F13^{iii}	0.99	2.16	3.04 (3)	147
C12—H12*B*···F16^{iii}	0.99	2.56	3.209 (17)	123
C13—H13*A*···F12^{iii}	0.99	2.52	3.33 (3)	139
C13—H13*A*···F13^{iv}	0.99	2.22	2.90 (3)	125
C13—H13*A*···F13^{iii}	0.99	2.42	3.25 (3)	142
C13—H13*B*···F15^{iv}	0.99	2.59	3.50 (3)	153
C20—H20*A*···O2*W*^{v}	0.98	2.27	3.14 (5)	148
C23—H23···F22^{v}	0.95	2.41	3.339 (19)	166
C27—H27*A*···F12^{vi}	0.99	2.41	3.17 (3)	134
C27—H27*A*···F14^{vi}	0.99	2.41	3.386 (14)	169
C27—H27*A*···F16^{vi}	0.99	2.5	3.283 (17)	136
C41—H41*A*···N60	0.98	2.59	3.416 (18)	142
C41—H41*B*···F31^{ii}	0.98	2.0	2.89 (3)	151
C41—H41*B*···F32^{ii}	0.98	2.43	3.25 (3)	140
C41—H41*C*···N54^{vii}	0.98	2.22	2.849 (15)	121
C53—H53*A*···F22^{viii}	0.99	2.56	3.14 (2)	117

C53—H53*A*···F23^viii	0.99	2.6	3.54 (3)	160
C55—H55*A*···F23	0.99	2.57	3.48 (3)	152
C56—H56*B*···F11^viii	0.99	2.58	3.288 (17)	129
C56—H56*B*···F14^viii	0.99	2.25	3.062 (15)	138
C56—H56*B*···F15^viii	0.99	2.0	2.97 (3)	166
C58—H58*A*···F14	0.99	2.39	3.253 (11)	145
C58—H58*A*···F16	0.99	2.6	3.438 (18)	142
C59—H59*A*···F31	0.99	2.52	3.18 (2)	124
C59—H59*A*···F33^ii	0.99	2.6	3.428 (17)	142
C59—H59*B*···F22^i	0.99	2.52	3.416 (19)	151
O1*W*—H1*W*···F15^viii	0.87 (1)	2.64 (7)	3.39 (3)	144 (10)
O1*W*—H2*W*···Cl02^vii	0.87 (1)	2.33 (2)	3.184 (12)	170 (9)
O2*W*—H3*W*···N60	0.93 (2)	2.52 (3)	3.40 (3)	160 (2)
N14—H14*A*···Cl02^ix	0.86 (1)	2.31 (3)	3.133 (6)	159 (8)
N14—H14*B*···N21^x	0.86 (1)	2.22 (4)	3.035 (9)	159 (8)
O41—H41···O2*W*	1.00 (5)	1.84 (6)	2.732 (9)	146 (10)
N54—H54*B*···Cl02	0.86 (1)	2.48 (4)	3.279 (6)	154 (7)
N57—H57*A*···Cl02	0.86 (1)	2.65 (4)	3.444 (7)	154 (7)
C56—H57*B*···O1*W*	1.37 (9)	2.41 (8)	3.66 (2)	151 (2)
N57—H57*B*···O1*W*	0.87 (1)	2.41 (8)	2.971 (13)	123 (7)
N60—H60*A*···O1*W*	0.86 (1)	2.55 (6)	3.185 (13)	132 (7)
N60—H60*A*···O2*W*	0.86 (1)	2.60 (5)	3.40 (3)	155 (7)

Symmetry codes: (i) -*x*, -*y*+1, -*z*; (ii) -*x*, -*y*+1, -*z*+1; (iii) -*x*+1, -*y*+1, -*z*+1; (iv) *x*, *y*-1, *z*+1; (v) -*x*+1, -*y*, -*z*+1; (vi) *x*+1, *y*-1, *z*+1; (vii) *x*-1, *y*, *z*; (viii) -*x*+1, -*y*+1, -*z*; (ix) -*x*+2, -*y*, -*z*+1; (x) -*x*+2, -*y*, -*z*+2.

Geometric parameters (Å, º) for (Co_tpa_PyPropHp)

C2—C3	1.348 (14)	C62—C63	1.390 (17)
C2—N1	1.400 (14)	C63—C64	1.40 (2)
C2—C7	1.461 (18)	C63—H63	0.9300
C3—O30	1.347 (11)	C64—C65	1.34 (2)
C3—C4	1.424 (15)	C64—H64	0.9300
C4—O40	1.265 (13)	C65—C66	1.352 (17)
C4—C5	1.433 (15)	C65—H65	0.9300
C5—C6	1.379 (17)	C66—N61	1.327 (15)
C5—H5	0.9300	C66—H66	0.9300
C6—N1	1.308 (16)	C70—C72	1.49 (2)
C6—H6	0.9300	C70—N5	1.497 (16)
C7—H7A	0.9600	C70—H70A	0.9700
C7—H7B	0.9600	C70—H70B	0.9700
C7—H7C	0.9600	C72—N71	1.321 (14)

C8—N1	1.472 (15)	C72—C73	1.426 (19)
C8—C9	1.486 (19)	C73—C74	1.33 (2)
C8—H8A	0.9700	C73—H73	0.9300
C8—H8B	0.9700	C74—C75	1.34 (2)
C9—C10	1.528 (19)	C74—H74	0.9300
C9—H9A	0.9700	C75—C76	1.379 (17)
C9—H9B	0.9700	C75—H75	0.9300
C10—N10	1.421 (19)	C76—N71	1.329 (15)
C10—H10A	0.9700	C76—H76	0.9300
C10—H10B	0.9700	N11—C12	1.3267
C14—C15	1.29 (5)	N11—C16	1.3962
C14—C13	1.36 (4)	C12—C13	1.3253
C14—H14	0.9300	C13—H13	0.9300
C17—N10	1.48 (2)	C15—C16	1.4724
C17—C12	1.55 (2)	C15—H15	0.9300
C17—H17A	0.9700	C16—H16	0.9300
C17—H17B	0.9700	N5—Co1	1.939 (10)
C50—C52	1.480 (18)	N10—H10	0.87 (3)
C50—N5	1.525 (16)	N51—Co1	1.909 (9)
C50—H50A	0.9700	N61—Co1	1.920 (9)
C50—H50B	0.9700	N71—Co1	1.928 (9)
C52—N51	1.372 (15)	O1W—H1A	0.86 (3)
C52—C53	1.39 (2)	O1W—H1B	0.92 (6)
C53—C54	1.41 (3)	O2W—H2A	0.864 (15)
C53—H53	0.9300	O2W—H2B	0.861 (15)
C54—C55	1.31 (2)	O3W—H3A	0.865 (15)
C54—H54	0.9300	O3W—H3B	0.862 (15)
C55—C56	1.364 (18)	O21—Cl2	1.26 (2)
C55—H55	0.9300	O22—Cl2	1.32 (2)
C56—N51	1.338 (14)	O30—Co1	1.883 (7)
C56—H56	0.9300	O31—Cl3	1.443 (11)
C60—N5	1.466 (14)	O32—Cl3	1.394 (11)
C60—C62	1.504 (17)	O33—Cl3	1.356 (12)
C60—H60A	0.9700	O34—Cl3	1.416 (12)
C60—H60B	0.9700	O40—Co1	1.903 (7)
C62—N61	1.350 (14)	O1OH—H1OH	0.97 (14)
C3—C2—N1	117.8 (10)	C65—C66—H66	120.6000
C3—C2—C7	121.7 (11)	C72—C70—N5	106.2 (9)
N1—C2—C7	120.5 (11)	C72—C70—H70A	110.5000
O30—C3—C2	122.5 (10)	N5—C70—H70A	110.5000

O30—C3—C4	114.0 (8)	C72—C70—H70B	110.5000
C2—C3—C4	123.5 (10)	N5—C70—H70B	110.5000
O40—C4—C3	120.4 (10)	H70A—C70—H70B	108.7000
O40—C4—C5	123.3 (10)	N71—C72—C73	118.6 (13)
C3—C4—C5	116.3 (10)	N71—C72—C70	115.4 (11)
C6—C5—C4	116.7 (10)	C73—C72—C70	125.9 (12)
C6—C5—H5	121.6000	C74—C73—C72	120.7 (14)
C4—C5—H5	121.7000	C74—C73—H73	119.6000
N1—C6—C5	125.3 (10)	C72—C73—H73	119.7000
N1—C6—H6	117.3000	C73—C74—C75	119.0 (13)
C5—C6—H6	117.4000	C73—C74—H74	120.5000
C2—C7—H7A	109.5000	C75—C74—H74	120.5000
C2—C7—H7B	109.5000	C74—C75—C76	120.0 (13)
H7A—C7—H7B	109.5000	C74—C75—H75	120.0000
C2—C7—H7C	109.5000	C76—C75—H75	120.0000
H7A—C7—H7C	109.5000	N71—C76—C75	121.1 (12)
H7B—C7—H7C	109.5000	N71—C76—H76	119.4000
N1—C8—C9	115.3 (11)	C75—C76—H76	119.4000
N1—C8—H8A	108.4000	C12—N11—C16	118.2000
C9—C8—H8A	108.4000	C13—C12—N11	125.8000
N1—C8—H8B	108.5000	C13—C12—C17	118.3 (13)
C9—C8—H8B	108.5000	N11—C12—C17	115.8 (13)
H8A—C8—H8B	107.5000	C12—C13—C14	117.9 (17)
C8—C9—C10	112.4 (12)	C12—C13—H13	121.0000
C8—C9—H9A	109.1000	C14—C13—H13	121.1000
C10—C9—H9A	109.1000	C14—C15—C16	121.7 (14)
C8—C9—H9B	109.1000	C14—C15—H15	119.1000
C10—C9—H9B	109.1000	C16—C15—H15	119.2000
H9A—C9—H9B	107.9000	N11—C16—C15	114.9000
N10—C10—C9	111.6 (13)	N11—C16—H16	122.5000
N10—C10—H10A	109.3000	C15—C16—H16	122.5000
C9—C10—H10A	109.3000	C6—N1—C2	120.2 (9)
N10—C10—H10B	109.3000	C6—N1—C8	117.8 (10)
C9—C10—H10B	109.3000	C2—N1—C8	121.9 (10)
H10A—C10—H10B	108.0000	C60—N5—C70	112.9 (9)
C15—C14—C13	121 (2)	C60—N5—C50	109.5 (10)
C15—C14—H14	119.6000	C70—N5—C50	110.9 (9)
C13—C14—H14	119.4000	C60—N5—Co1	112.2 (7)
N10—C17—C12	111.8 (12)	C70—N5—Co1	106.0 (8)
N10—C17—H17A	109.3000	C50—N5—Co1	105.0 (7)
C12—C17—H17A	109.3000	C10—N10—C17	112.9 (16)

N10—C17—H17B	109.3000	C10—N10—H10	150 (10)
C12—C17—H17B	109.3000	C17—N10—H10	95 (10)
H17A—C17—H17B	107.9000	C56—N51—C52	119.1 (10)
C52—C50—N5	106.6 (9)	C56—N51—Co1	128.3 (8)
C52—C50—H50A	110.4000	C52—N51—Co1	112.4 (8)
N5—C50—H50A	110.4000	C66—N61—C62	121.9 (10)
C52—C50—H50B	110.4000	C66—N61—Co1	124.9 (8)
N5—C50—H50B	110.4000	C62—N61—Co1	113.1 (7)
H50A—C50—H50B	108.6000	C72—N71—C76	120.4 (10)
N51—C52—C53	118.1 (13)	C72—N71—Co1	113.2 (8)
N51—C52—C50	114.7 (10)	C76—N71—Co1	126.0 (8)
C53—C52—C50	127.2 (13)	H1A—O1W—H1B	130 (10)
C52—C53—C54	120.3 (16)	H2A—O2W—H2B	121 (10)
C52—C53—H53	119.8000	H3A—O3W—H3B	120 (3)
C54—C53—H53	119.9000	C3—O30—Co1	109.2 (6)
C55—C54—C53	119.4 (14)	C4—O40—Co1	108.4 (7)
C55—C54—H54	120.3000	O21^{i}—Cl2—O21	126 (3)
C53—C54—H54	120.2000	O21^{i}—Cl2—O22	109 (2)
C54—C55—C56	119.6 (15)	O21—Cl2—O22	104.4 (18)
C54—C55—H55	120.2000	O21^{i}—Cl2—O22^{i}	104.4 (18)
C56—C55—H55	120.2000	O21—Cl2—O22^{i}	109 (2)
N51—C56—C55	123.4 (13)	O22—Cl2—O22^{i}	103 (3)
N51—C56—H56	118.3000	O33—Cl3—O32	111.4 (8)
C55—C56—H56	118.3000	O33—Cl3—O34	108.4 (12)
N5—C60—C62	110.3 (9)	O32—Cl3—O34	108.1 (9)
N5—C60—H60A	109.6000	O33—Cl3—O31	111.7 (10)
C62—C60—H60A	109.6000	O32—Cl3—O31	108.2 (7)
N5—C60—H60B	109.6000	O34—Cl3—O31	108.9 (9)
C62—C60—H60B	109.6000	O30—Co1—O40	87.4 (3)
H60A—C60—H60B	108.1000	O30—Co1—N51	93.5 (4)
N61—C62—C63	120.8 (12)	O40—Co1—N51	91.2 (4)
N61—C62—C60	117.4 (9)	O30—Co1—N61	94.1 (3)
C63—C62—C60	121.8 (11)	O40—Co1—N61	178.5 (4)
C62—C63—C64	116.1 (13)	N51—Co1—N61	88.9 (4)
C62—C63—H63	122.0000	O30—Co1—N71	97.1 (4)
C64—C63—H63	122.0000	O40—Co1—N71	89.5 (4)
C65—C64—C63	120.5 (13)	N51—Co1—N71	169.4 (4)
C65—C64—H64	119.7000	N61—Co1—N71	90.2 (4)
C63—C64—H64	119.7000	O30—Co1—N5	178.9 (4)
C64—C65—C66	121.9 (14)	O40—Co1—N5	92.0 (3)
C64—C65—H65	119.1000	N51—Co1—N5	85.6 (4)

C66—C65—H65	119.0000	N61—Co1—N5	86.5 (4)
N61—C66—C65	118.8 (13)	N71—Co1—N5	83.8 (4)
N61—C66—H66	120.6000		
N1—C2—C3—O30	-179.7 (9)	C5—C6—N1—C8	173.9 (12)
C7—C2—C3—O30	1.6 (16)	C3—C2—N1—C6	1.3 (15)
N1—C2—C3—C4	2.1 (15)	C7—C2—N1—C6	-180.0 (12)
C7—C2—C3—C4	-176.6 (11)	C3—C2—N1—C8	-174.4 (11)
O30—C3—C4—O40	-3.5 (14)	C7—C2—N1—C8	4.3 (17)
C2—C3—C4—O40	174.8 (10)	C9—C8—N1—C6	-91.6 (15)
O30—C3—C4—C5	177.0 (9)	C9—C8—N1—C2	84.2 (15)
C2—C3—C4—C5	-4.7 (15)	C62—C60—N5—C70	-122.5 (11)
O40—C4—C5—C6	-175.6 (11)	C62—C60—N5—C50	113.3 (11)
C3—C4—C5—C6	3.9 (14)	C62—C60—N5—Co1	-2.8 (14)
C4—C5—C6—N1	-0.8 (18)	C72—C70—N5—C60	82.4 (12)
N1—C8—C9—C10	67.6 (16)	C72—C70—N5—C50	-154.2 (9)
C8—C9—C10—N10	58.9 (15)	C72—C70—N5—Co1	-40.8 (10)
N5—C50—C52—N51	-33.3 (13)	C52—C50—N5—C60	-79.3 (11)
N5—C50—C52—C53	145.2 (13)	C52—C50—N5—C70	155.4 (10)
N51—C52—C53—C54	-4 (2)	C52—C50—N5—Co1	41.3 (10)
C50—C52—C53—C54	177.1 (13)	C9—C10—N10—C17	172.1 (11)
C52—C53—C54—C55	2 (2)	C12—C17—N10—C10	73.6 (18)
C53—C54—C55—C56	1 (2)	C55—C56—N51—C52	-1.1 (18)
C54—C55—C56—N51	-1 (2)	C55—C56—N51—Co1	172.3 (10)
N5—C60—C62—N61	7.7 (16)	C53—C52—N51—C56	4.0 (17)
N5—C60—C62—C63	-174.8 (12)	C50—C52—N51—C56	-177.3 (10)
N61—C62—C63—C64	1.7 (18)	C53—C52—N51—Co1	-170.5 (10)
C60—C62—C63—C64	-175.7 (13)	C50—C52—N51—Co1	8.2 (12)
C62—C63—C64—C65	-1 (2)	C65—C66—N61—C62	2.4 (17)
C63—C64—C65—C66	0 (2)	C65—C66—N61—Co1	-173.4 (10)
C64—C65—C66—N61	-1 (2)	C63—C62—N61—C66	-2.7 (17)
N5—C70—C72—N71	31.1 (13)	C60—C62—N61—C66	174.8 (11)
N5—C70—C72—C73	-149.9 (11)	C63—C62—N61—Co1	173.5 (9)
N71—C72—C73—C74	1.4 (18)	C60—C62—N61—Co1	-8.9 (13)
C70—C72—C73—C74	-177.6 (12)	C73—C72—N71—C76	1.5 (15)
C72—C73—C74—C75	-3 (2)	C70—C72—N71—C76	-179.4 (10)
C73—C74—C75—C76	2 (2)	C73—C72—N71—Co1	175.5 (9)
C74—C75—C76—N71	0.8 (19)	C70—C72—N71—Co1	-5.4 (12)
C16—N11—C12—C13	-0.9000	C75—C76—N71—C72	-2.7 (16)
C16—N11—C12—C17	-177.1 (12)	C75—C76—N71—Co1	-175.8 (9)
N10—C17—C12—C13	-121.0 (15)	C2—C3—O30—Co1	-170.5 (8)

N10—C17—C12—N11	55.5 (19)	C4—C3—O30—Co1	7.8 (10)
N11—C12—C13—C14	7.4 (17)	C3—C4—O40—Co1	-2.8 (11)
C17—C12—C13—C14	-176.5 (19)	C5—C4—O40—Co1	176.7 (8)
C15—C14—C13—C12	-9 (4)	C3—O30—Co1—O40	-7.5 (6)
C13—C14—C15—C16	5 (4)	C3—O30—Co1—N51	83.5 (7)
C12—N11—C16—C15	-3.7000	C3—O30—Co1—N61	172.6 (7)
C14—C15—C16—N11	2 (2)	C3—O30—Co1—N71	-96.7 (7)
C5—C6—N1—C2	-2.0 (19)		

Symmetry code: (i) -*x*+1, -*y*+1, *z*.

Hydrogen-bond geometry (Å, º) for (Co_tpa_PyPropHp)

D—H···*A*	*D*—H	H···*A*	*D*···*A*	*D*—H···*A*
C8—H8*A*···O32	0.97	2.61	3.203 (19)	120
C17—H17*A*···O32^{ii}	0.97	2.57	3.35 (2)	137
C50—H50*B*···O33^{iii}	0.97	2.37	3.286 (18)	157
C55—H55···O21^{iv}	0.93	2.58	3.13 (3)	118
C56—H56···O30	0.93	2.55	3.014 (14)	111
C60—H60*A*···O2*W*	0.97	2.37	3.31 (3)	163
C60—H60*B*···O1*W*	0.97	2.60	3.50 (2)	154
C63—H63···O3*W*^{v}	0.93	2.64	3.50 (3)	154
C66—H66···O30	0.93	2.44	2.923 (13)	112
C70—H70*A*···O31^{iii}	0.97	2.48	3.423 (17)	166
C70—H70*B*···O1*OH*	0.97	2.42	3.33 (3)	157
C76—H76···O30	0.93	2.65	3.099 (13)	110
O1*W*—H1*A*···O3*W*^{v}	0.86 (3)	2.4 (2)	3.01 (4)	130 (20)
O1*W*—H1*B*···O1*OH*	0.92 (6)	1.49 (10)	2.40 (4)	170 (20)
O1*OH*—H1*OH*···O1*W*^{vi}	0.97 (14)	1.78 (11)	2.72 (4)	160 (40)
O2*W*—H2*A*···O1*OH*^{vi}	0.86 (2)	1.85 (11)	2.67 (4)	160 (20)
O2*W*—H2*B*···N11^{vii}	0.86 (2)	2.01 (3)	2.87 (2)	180 (20)
O3*W*—H3*A*···O1*W*^{viii}	0.87 (2)	2.22 (9)	3.01 (4)	153 (18)

Symmetry codes: (ii) *x*, *y*, *z*-1; (iii) *x*+1/4, -*y*+3/4, *z*-1/4; (iv) -*x*+1, -*y*+1, *z*-1; (v) *x*+1/4, -*y*+5/4, *z*+1/4; (vi) -*x*+3/2, -*y*+1, *z*-1/2; (vii) -*x*+5/4, *y*+1/4, *z*+1/4; (viii) *x*-1/4, -*y*+5/4, *z*-1/4.

Document origin: *publCIF* [Westrip, S. P. (2010). *J. Apply. Cryst.*, **43**, 920-925].
